# An algorithm for nonlinear problems with variational inequality methods

**DOI:** 10.1371/journal.pone.0329105

**Published:** 2025-08-28

**Authors:** Huaping Huang, Imo Kalu Agwu, Umar Ishtiaq, Ibrahim Alraddadi, Kashif Saleem, Ioannis K. Argyros

**Affiliations:** 1 School of Mathematics and Statistics, Chongqing Three Gorges University, Wanzhou, China; 2 Department of Mathematics, Micheal Okpara University of Agriculture, Umudike, Umuahia Abia State, Nigeria; 3 Office of Research, Innovation and Commercialization, University of Management and Technology, Lahore, Pakistan; 4 Department of Mathematics, Faculty of Science, Islamic University of Madinah, Madinah, Saudi Arabia; 5 School of Computing, Macquarie University, North Ryde, New South Wales, Australia; 6 Department of Computing and Mathematical Sciences, Cameron University, Lawton, Oklahoma, United States of America; King Abdulaziz University Faculty of Sciences, SAUDI ARABIA

## Abstract

The purpose of this paper is to introduce a mapping in the framework of a 2-uniformly smooth and uniformly convex Banach space and obtain a common solution for the fixed point set of finite families of $\beta_{i}$-enriched non-expansive mappings and (Ψ,$\beta_{i}$)-enriched strictly pseudocontractive mappings. Two solution sets of variational inequality problems are also given. Moreover, we prove several strong convergence theorems for the fixed point set of the finite family of (Ψ,$\beta_{i}$)-enriched strictly pseudocontractive mappings and the solution set of variational inequality problems. By using the famous Mann-Halpern type iterative method we obtain some strong convergence theorems. Our results from this paper improve and generalize many well-known results in the existing literature.

## 1 Introduction and preliminaries

Throughout this paper, $E,C,E^{⋆},UCB,USB$ and ℝ will represent the real Banach space, a nonempty subset of E, the topological duala space of E, uniformly convex, uniformly smooth Banach spaces and the set of real numbers, respectively. Let *q* > 1 be a constant and define the mapping $J_{q}:E⟶2^{E^{⋆}}$ by

$$J_{q}(\ell )=\{\ell^{⋆}\in E^{⋆}:\langle \ell ,\;\ell^{⋆}\rangle =\|\ell {\|}^{q}\;and\;\|\ell^{⋆}\|=\|\ell {\|}^{q-1},\forall \ell \in E\}.$$ (1.1)

Then, $J_{q}$ is called generalized duality map on E. In particular, $J_{2}$ is called normalized duality mapping and it is often represented by J. It has been established (see, for instance, [1]) that $J_{q}(\ell )=\|\ell {\|}^{q-2}J(\ell )$ if ℓ≠0 and that if $E^{⋆}$ is strictly convex, then $J_{q}$ is single-valued, which is denoted by *j*_*q*_, but it becomes an identity operator in Hilbert spaces. For convenience, we shall use *J* in place of J where necessary.

**Definition 1.1.** Let E be Banach space. Then

1. E is called *UCB* if given *ε* with 0<ε≤2, the inequalities $\|\ell \|\leq 1,\|\varpi^{o}\|\leq 1$ and $\|\ell \;-\;\varpi^{o}\|\geq \varepsilon$ entail that we can find δ>0 such that $\|\frac{\ell +\varpi^{o}}{2}\|\leq 1-\delta$.
2. E is called smooth if for each $\ell \in {𝑆}_{E}=\{\ell \in E:\|\ell \|=1\}$, we can find a unique function $j_{\ell }\in E^{⋆}$ such that $\langle \ell ,j_{\ell }\rangle =\|\ell \|$ and $\|j_{\ell }\|=1$.
3. E is called *q*-*USB* (*q* > 1) if we can find a fixed constant C>0 such that $\rho_{E}(\gamma )\leq C\gamma^{q}$, $\forall \gamma \in R^{+}$ ($R^{+}$ denotes the set of positive real numbers). It is not difficult to see that every *q*-USB is uniformly smooth.

**Definition 1.2.** Let E be a Banach space. Then, a function $\rho_{E}:R^{+}⟶R^{+}$ is called modulus of smoothness of E if the following identity holds:


$$\rho_{E}(\gamma )=\sup\{\frac{\|\ell +\varpi^{o}\|+\|\ell -\varpi^{o}\|}{2}-1:\|\ell \|=1,\|\varpi^{o}\|=\gamma \}.$$

E is called uniformly smooth if


$$\lim_{\gamma \to 0}\frac{\rho_{E}(\gamma )}{\gamma }=0.$$

It has been established that if E is a *USB* then it is a smooth Banach space.

Suppose that ℑ:C⟶C is a nonlinear mapping. Then, we refer to F(ℑ),N and R as the set of fixed point of ℑ, the the set of positive integers and the set of real numbers, respectively. When $\left\{\ell_{n}\right\}$ is a sequence in C, strong (respectively weak) convergence to a point ℓ will be represented with → (resp. ⇀).

**Definition 1.3.** ℑ is called (Ψ,ϑ)-enriched strictly pseudocontractive mapping (*ESPM*) (see [2,3]) if $\forall \ell ,\varpi^{o}\in C$, ∃Ψ∈[0,∞) and ϑ∈[0,1) such that


$$\|\Psi (\ell -\varpi^{o})+ℑ\ell -ℑ\varpi^{o}{\|}^{2}\leq (\Psi +1{)}^{2}\|\ell -\varpi^{o}{\|}^{2}+\vartheta \|\ell -\varpi^{o}-(ℑ\ell -ℑ\varpi^{o}){\|}^{2},$$

or equivalently,

$$\langle \Psi (\ell -\varpi^{o})+ℑ\ell -ℑ\varpi^{o},j((\Psi +1)(\ell -\varpi^{o}))\rangle \leq (\Psi +1{)}^{2}\|\ell -\varpi^{o}{\|}^{2}-\vartheta \|\ell -\varpi^{o}-(ℑ\ell -ℑ\varpi^{o}){\|}^{2},$$ (1.2)

where $j(\ell -\varpi^{o})\in J(\ell -\varpi^{o})$. Set $\Psi =\frac{1}{\omega }-1$ in (1.2). Then, by Proposition 1.1 (3) (see below), it is easy to see that

$$\langle {ℑ}_{\omega }\ell -{ℑ}_{\omega }\varpi^{o},j(\ell -\varpi^{o})\rangle \leq \|\ell -\varpi^{o}{\|}^{2}-\vartheta \|\ell -\varpi^{o}-({ℑ}_{\omega }\ell -{ℑ}_{\omega }\varpi^{o}){\|}^{2},$$ (1.3)

where ${ℑ}_{\omega }=(1-\omega )I+\omega ℑ$ is an average operator and *I* denotes the identity mapping, and ${ℑ}_{\omega }$ is called ϑ-strictly pseudocontractive. The inequality (1.3) could be written equivalently as

$$\langle (I-{ℑ}_{\omega })\ell -(I-{ℑ}_{\omega })\varpi^{o},j(\ell -\varpi^{o})\rangle \geq \vartheta \|\ell -\varpi^{o}-({ℑ}_{\omega }\ell -{ℑ}_{\omega }\varpi^{o}){\|}^{2}.$$ (1.4)

**Remark 1.1.** Set ϑ=0, then (1.2) becomes

$$\langle \Psi (\ell -\varpi^{o})+ℑ\ell -ℑ\varpi^{o},j((\Psi +1)(\ell -\varpi^{o}))\rangle \leq (\Psi +1{)}^{2}\|\ell -\varpi^{o}{\|}^{2},$$ (1.5)

which defines the class of mappings known as Ψ-enriched non-expansive mapping (Ψ-ENM), which was first considered by Berinde [4,5]. This class of mappings contains one of the initial classes of mappings (nonexpansive mappings) whose fixed points were evaluated using geometric properties instead of the compactness conditions. Aside generalizing the class of contraction mappings (and its intimate relationship with the monotonicity techniques), nonexpansive mapping practically stands as transition operators for initial value problems of differential inclusion, accretive operators, monotone operators, equilibrium problems, and variational inequality problems. Recently, several extensions and generalizations of this important class of mappings have been examined by different researchers; see, for instance, [6–8] and the references contained in them.

**Example 1.1.** (See [3]) Let $E={\mathbb{R}}^{2}$ be equipped with the Euclidean norm and


$$C=\{(\ell_{1},\ell_{2})\in {\mathbb{R}}^{2}:\ell_{1},\ell_{2}\geq 0,\ell_{1}^{2}+\ell_{2}^{2}\leq 1\}.$$

Define the mapping ℑ:C⟶C by $ℑ(\varpi^{o},\ell )=(\frac{\varpi^{o}}{2},\frac{\ell }{2}).$ Let Ψ∈[0,+∞) and ϑ∈[0,1). Then, for all $\varpi^{o},\ell \in C$, we have


$$∥\Psi (\varpi^{o}-\ell )+ℑ\varpi^{o}-ℑ\ell {∥}^{2}=∥\Psi [(\varpi_{1}^{o},\ell_{1})-(\varpi_{2}^{o},\ell_{2})]+ℑ(\varpi_{1}^{o},\ell_{1})-ℑ(\varpi_{2}^{o},\ell_{2}){∥}^{2}$$

$$=\|\Psi (\varpi_{1}^{o}-\varpi_{2}^{o},\ell_{1}-\ell_{2})+(\frac{\varpi_{1}^{o}}{2},\frac{\ell_{1}}{2})-(\frac{\varpi_{2}^{o}}{2},\frac{\ell_{2}}{2}){\|}^{2}$$

$$=\|(\Psi (\varpi_{1}^{o}-\varpi_{2}^{o})+\frac{\varpi_{1}^{o}-\varpi_{2}^{o}}{2},\Psi (\ell_{1}-\ell_{2})+\frac{\ell_{1}-\ell_{2}}{2}){\|}^{2}$$

$$=\|(\frac{2\Psi \varpi_{1}^{o}-2\Psi \varpi_{2}^{o}+\varpi_{1}^{o}-\varpi_{2}^{o}}{2},\frac{2\Psi \ell_{1}-2\Psi \ell_{2}+\ell_{1}-\ell_{2}}{2}){\|}^{2}$$

$$=\|(\frac{(2\Psi +1)\varpi_{1}^{o}-(2\Psi +1)\varpi_{2}^{o}}{2},\frac{(2\Psi +1)\ell_{1}-(2\Psi +1)\ell_{2}}{2}){\|}^{2}$$

$$=(\frac{2\Psi +1}{2}{)}^{2}\|\left(\varpi_{1}^{o}-\varpi_{2}^{o},\ell_{1}-\ell_{2}\right){\|}^{2}$$

$$=(\frac{2\Psi +1}{2}{)}^{2}\|\left(\varpi_{1}^{o},\ell_{1})-(\varpi_{2}^{o},\ell_{2}\right){\|}^{2}.$$ (1.6)

Also, by setting $Q=(\Psi +1{)}^{2}∥\varpi^{o}-\ell {∥}^{2}+\vartheta ∥\varpi^{o}-\ell -(ℑ\varpi^{o}-ℑ\ell ){∥}^{2}$, we obtain that


$$Q=(\Psi +1{)}^{2}∥(\varpi_{1}^{o},\ell_{1})-(\varpi_{2}^{o},\ell_{2}){∥}^{2}+\vartheta \|(\varpi_{1}^{o},\ell_{1})-(\varpi_{2}^{o},\ell_{2})-[(\frac{\varpi_{1}^{o}}{2},\frac{\ell_{1}}{2})-(\frac{\varpi_{2}^{o}}{2},\frac{\ell_{2}}{2})]{\|}^{2}$$

$$=(\Psi +1{)}^{2}∥(\varpi_{1}^{o},\ell_{1})-(\varpi_{2}^{o},\ell_{2}){∥}^{2}+\vartheta \|(\varpi_{1}^{o}-\varpi_{2}^{o},\ell_{1}-\ell_{2})-(\frac{\varpi_{1}^{o}-\varpi_{2}^{o}}{2},\frac{\ell_{1}-\ell_{2}}{2}){\|}^{2}$$

$$=(\Psi +1{)}^{2}∥(\varpi_{1}^{o},\ell_{1})-(\varpi_{2}^{o},\ell_{2}){∥}^{2}+\vartheta \|(\varpi_{1}^{o}-\varpi_{2}^{o}-\frac{\varpi_{1}^{o}-\varpi_{2}^{o}}{2},\ell_{1}-\ell_{2}-\frac{\ell_{1}-\ell_{2}}{2}){\|}^{2}$$

$$=(\Psi +1{)}^{2}∥(\varpi_{1}^{o},\ell_{1})-(\varpi_{2}^{o},\ell_{2}){∥}^{2}+\vartheta \|(\frac{2\varpi_{1}^{o}-2\varpi_{2}^{o}-\varpi_{1}^{o}+\varpi_{2}^{o}}{2},\frac{2\ell_{1}-2\ell_{2}-\ell_{1}+\ell_{2}}{2}){\|}^{2}$$

$$=(\Psi +1{)}^{2}∥(\varpi_{1}^{o},\ell_{1})-(\varpi_{2}^{o},\ell_{2}){∥}^{2}+\vartheta \|(\frac{\varpi_{1}^{o}-\varpi_{2}^{o}}{2},\frac{\ell_{1}-\ell_{2}}{2}){\|}^{2}$$

$$=(\Psi +1{)}^{2}∥(\varpi_{1}^{o},\ell_{1})-(\varpi_{2}^{o},\ell_{2}){∥}^{2}+\frac{\vartheta }{2}\|(\varpi_{1}^{o}-\varpi_{2}^{o},\ell_{1}-\ell_{2}){\|}^{2}$$

$$=(\Psi +1{)}^{2}∥(\varpi_{1}^{o},\ell_{1})-(\varpi_{2}^{o},\ell_{2}){∥}^{2}+\frac{\vartheta }{2}\|(\varpi_{1}^{o},\ell_{1})-(\varpi_{2}^{o},\ell_{2}){∥}^{2}$$

$$=[(\Psi +1{)}^{2}+\frac{\vartheta }{2}]\|(\varpi_{1}^{o},\ell_{1})-(\varpi_{2}^{o},\ell_{2}){\|}^{2}.$$ (1.7)

(1.6) and (1.7) imply


$$∥\Psi (\varpi^{o}-\ell )+ℑ\varpi^{o}-ℑ\ell {∥}^{2}=(\frac{2\Psi +1}{2}{)}^{2}∥\left(\varpi_{1}^{o},\ell_{1})-(\varpi_{2}^{o},\ell_{2}\right){∥}^{2}$$

$$\leq [(\Psi +1{)}^{2}+\frac{\vartheta }{2}]∥(\varpi_{1}^{o},\ell_{1})-(\varpi_{2}^{o},\ell_{2}){∥}^{2}$$

$$=(\Psi +1{)}^{2}∥\varpi^{o}-\ell {∥}^{2}+\vartheta ∥\varpi^{o}-\ell -(ℑ\varpi^{o}-ℑ\ell ){∥}^{2}.$$

Thus, the mapping ℑ is a (Ψ,ϑ)-enriched strictly pseudocontractive mapping. Further, observe that (0,0) is a unique fixed point of ℑ.

In the setup of a real Hilbert space, inequality (1.3) is written as

$$\|{ℑ}_{\omega }\ell -{ℑ}_{\omega }\varpi^{o}{\|}^{2}\leq \|\ell -\varpi^{o}{\|}^{2}+\vartheta \|\ell -\varpi^{o}-({ℑ}_{\omega }\ell -{ℑ}_{\omega }\varpi^{o}){\|}^{2},$$ (1.8)

where ${ℑ}_{\omega }$ is described in inequality (1.3). Set ϑ=1 in (1.8), then we have the class of pseudocontractions.

It is worth noting that the class of (Ψ,ϑ)-ESPM was initiated by Berinde [2], as a generalization of the class of ϑ-strictly pseudocontractions (Recall that ℑ is known as ϑ-strictly pseudocontraction if $\|ℑ\ell -ℑ\varpi^{o}{\|}^{2}\leq \|\ell -\varpi^{o}{\|}^{2}+\vartheta \|\ell -\varpi^{o}-(ℑ\ell -ℑ\varpi^{o}){\|}^{2},$ for all $\ell ,\varpi^{o}\in C$). In [2], Berinde proved that every (Ψ,k)-enriched strictly pseudocontractive mapping defined on a bounded, closed and convex subset C of a real Hilbert space has a fixed point.

Let E be a Banach space, 0/≠C⊂E and 𝐷⊂C. A mapping 𝑃:C⟶𝐷 is sunny (see [10]) as long as 𝑃(ℓ+γ(ℓ−𝑃(ℓ)))=𝑃(ℓ), ∀ℓ∈C and γ≥0, whenever ℓ+γ(ℓ−𝑃(ℓ))∈C. *P* is called a retraction if 𝑃ℓ=ℓ and the sunny nonexpansive retraction (*SNR*) from C onto *D* if *P* is a retraction from C onto *D*.

Let 𝐴:C⟶E be a nonlinear operator. Then, *A* is called an accretive if we can find $j(\ell \;-\;\varpi^{o})\in J(\ell -\varpi^{o})$ such that


$$\langle 𝐴\ell -𝐴\varpi^{o},j(\ell -\varpi^{o})\rangle \geq 0,\forall \ell ,\varpi^{o}\in C.$$

*A* is called *α*-inverse strongly accretive if we can find $j(\ell -\varpi^{o})\in J(\ell -\varpi^{o})$ and α>0 such that


$$\langle 𝐴\ell -𝐴\varpi^{o},j(\ell -\varpi^{o})\rangle \geq \alpha \|𝐴\ell -𝐴\varpi^{o}{\|}^{2},\forall \ell ,\varpi^{o}\in C.$$

Note that if ℑ is a ϑ-strictly pseudocontractive mapping, then I−ℑ is ϑ-inverse strongly accretive.

Let E and C be described as above. Then, the variational inequality in E is to search for an $\ell^{⋆}\in C$ that assures the inequality

$$\langle 𝐴\ell^{⋆},j(\ell -\ell^{⋆})\geq 0,\forall \ell \in C$$ (1.9)

for some $j(\ell -\varpi^{o})\in J(\ell -\varpi^{o})$. Problem (1.9) was studied by Aoyama et al. [11]. The set of solutions of the problem (1.9) in E will be represented with S(C,𝐴), that is,

S(C,𝐴)={s∈C:⟨𝐴s,J(t−s)⟩≥0,∀t∈C}. (1.10)

Many practically oriented problems in pure and applied sciences, physics and economics can be recast in the form of (1.9); see, for example, [12–14]. To solve the problem (1.9), several researchers have attempted to use the following methods. Let ℑ be a nonexpansive mapping with a fixed point and the sequence $\{\alpha_{n}{\}}_{n\geq 0}$ be chosen such that $\left\{\alpha_{n}{\}}_{n\geq 0}\subset (0,1\right)$ and $\sum_{n=0}^{\infty }\alpha_{n}(1-\alpha_{n})=\infty$. Then the normal Mann iterative sequence $\{\ell_{n}{\}}_{n=0}^{\infty }$, first studied by Mann [15], is given by

$$\left\{ \begin{array}{c} \ell_{0}\in C,\\ \ell_{n+1}=(1-\alpha_{n})\ell_{n}+\alpha_{n}ℑ\ell_{n}. \end{array}\right.$$ (1.11)

The above sequence is known to admit weak convergence. In a quest to obtain strong convergence, Halpern [16], in 1967, initiated the following technique:

$$\left\{ \begin{array}{c} \ell_{0}\in C,\\ \ell_{n+1}=(1-\alpha_{n})\ell_{0}+\alpha_{n}ℑ\ell_{n}, \end{array}\right.$$ (1.12)

where ℑ is a nonexpansive mapping with a fixed point and $\left\{\alpha_{n}{\}}_{n\geq 0}\subset (0,1\right)$. He noted that the conditions $\lim_{n\to \infty }\alpha_{n}=0$ and $\sum_{n=0}^{\infty }\alpha_{n}=\infty$ are necessary in order to establish strong convergence of $\{\ell_{n}{\}}_{n\geq 0}$ to the fixed point of ℑ. Subsequently, several authors have modified the iteration technique (1.12) in an attempt to establish strong convergence results; see, for example, [17] and the references therein.

In [18], Zhou obtained strong convergence results using modified Mann-Halpern iteration technique for ϑ-strictly pseudocontractive mappings in a real 2-uniformly smooth Banach space.

In [11], Aoyama et al. studied Mann-type recursive sequence and showed that such a method admits weak convergence results for the class of nonexpansive mappings. In addition, they proved weak convergence theorem for finding the solution of the variational inequality problem (1.9) in a 2-uniformly smooth Banach space.

In [26], Karntunykarn and Suantai gave the following definition.

**Definition 1.4.** Let E be a real Banach space, 0/≠K⊂E and $\{{ℑ}_{r}{\}}_{r=1}^{m}:K⟶K$ be a finite family of mapping, where m∈N. Let $\delta_{r}=(\delta_{1}^{r},\delta_{2}^{r},\delta_{3}^{r})\in J\times J\times J$ for each r=1,2,⋯,m, where J=[0,1] and $\delta_{1}^{r}+\delta_{2}^{r}+\delta_{3}^{r}=1$. The mapping S:K⟶K given by


$$G_{0}=I$$

$$G_{1}=\delta_{1}^{1}{ℑ}_{1}G_{0}+\delta_{2}^{1}G_{0}+\delta_{3}^{1}I$$

$$G_{2}=\delta_{1}^{2}{ℑ}_{2}G_{1}+\delta_{2}^{2}G_{1}+\delta_{3}^{2}I$$

$$G_{3}=\delta_{1}^{3}{ℑ}_{3}G_{2}+\delta_{2}^{3}G_{2}+\delta_{3}^{3}I$$

⋮

$$G_{m-1}=\delta_{1}^{N-1}{ℑ}_{m-1}G_{m-2}+\delta_{2}^{N-1}G_{m-2}+\delta_{3}^{N-1}I$$

$$S=G_{m}=\delta_{1}^{m}{ℑ}_{m}G_{m-1}+\delta_{3}^{m}G_{m-1}+\delta_{3}^{m}I.$$ (1.13)

is known as the S-mapping generated from ${ℑ}_{1},{ℑ}_{2},\cdots ,{ℑ}_{m}$ and $\delta_{1},\delta_{2},\cdots ,\delta_{m}$

If $\delta_{3}^{i}=0$, for every r=1,2,⋯,m, then (1.13) reduces to the class of *K*-mapping which was first studied in [19].

In [20], by means of *K*-mapping defined in a real 2-uniformly smooth Banach space, Karntunykarn and Suantai constructed the Mann-type iterative method for finding a common element of the sets of fixed points of an ϑ-strictly pseudocontractive mapping and nonexpansive mapping, and the set of solutions of finite family of variational inequality problems.

Recently, Karntunykarn [10] introduced the mapping below as a generalization of (1.13).

**Definition 1.5.** Let E be a real Banach space, 0/≠K⊂E and $\{{ℑ}_{r}{\}}_{r=1}^{m},\{{ℑ}_{r}{\}}_{r=1}^{m}:K⟶K$ be finite families of mappings, where m∈N. Let $\delta_{r}=(\delta_{1}^{r},\delta_{2}^{r},\delta_{3}^{r})\in J\times J\times J$ for each r=1,2,⋯,m, where J=[0,1] and $\delta_{1}^{r}+\delta_{2}^{r}+\delta_{3}^{r}=1$. The mapping $D^{A}:K⟶K$ defined by


$$G_{0}={ℑ}_{1}=I$$

$$G_{1}={ℑ}_{1}(\delta_{1}^{1}{ℑ}_{1}G_{0}+\delta_{2}^{1}G_{0}+\delta_{3}^{1}I)$$

$$G_{2}={ℑ}_{2}(\delta_{1}^{2}{ℑ}_{2}G_{1}+\delta_{2}^{2}G_{1}+\delta_{3}^{2}I)$$

$$G_{3}={ℑ}_{3}(\delta_{1}^{3}{ℑ}_{3}G_{2}+\delta_{2}^{3}G_{2}+\delta_{3}^{3}I)$$ (1.14)

⋮

$$G_{m-1}={ℑ}_{m-1}(\delta_{1}^{m-1}{ℑ}_{m-1}G_{m-2}+\delta_{2}^{m-1}G_{m-2}+\delta_{3}^{N-1}I)$$

$$D=G_{m}={ℑ}_{m}(\delta_{1}^{m}{ℑ}_{N}G_{m-1}+\delta_{2}^{m}G_{m-1}+\delta_{3}^{m}I).$$

is known as ${\text{S}}^{\text{A}}$-mapping developed from ${ℑ}_{1},{ℑ}_{2},\cdots ,{ℑ}_{m},{ℑ}_{1},{ℑ}_{2},\cdots ,{ℑ}_{m}$ and $\delta_{1},\delta_{2},\cdots ,\delta_{m}$.

In addition, he established that ${\text{S}}^{\text{A}}$ mapping is nonexpansive and further showed that the Mann-Halpern-type algorithm can assure strong convergence results to the common element of the fixed point sets of a finite family of $\beta_{i}$-strictly pseudocontractive mappings and nonexpansive mappings and the two sets of variational inequality problems in a 2-uniformly smooth and uniformly convex Banach space.

After reviewing the above papers, it becomes pertinent to ask the questions below.

**Question 1.1.**

1. Is it possible to construct a mapping that could obtain a common element of the set of solutions of the variational inequality problem and fixed point problems of a finite family of $\left(\Psi_{i},\vartheta \right)$-ESPM and a finite family of $\Psi_{i}$-ENM?
2. Can the results established in [10] be extended to the classes of *β*-enriched non-expansive mappings and (Ψ,β)-enriched strictly pseudocontractive mappings?

Following the results in [10] , the purpose of this paper is to study the Mann-Halpern type method for the classes of a finite family of ϰ-ENM and $\left(\varkappa ,\vartheta_{i}\right)$-ESPM and variational inequality problems under a more general setting.

The rest of the paper is structured as follows: we present basic definitions and lemmas in Sect 2; in Sect 3, we present the algorithm for obtaining an answer to Question 1.1 and further analyze the convergence of the suggested method.

We need the following known results in the sequel. Meanwhile, E,C,UCB and *USB* are still as described in Sect Sect 1.

**Lemma 1.1.** *[21] Let E be a real 2-USB and UCB with the best smooth constant *B**^*2*^*, then the inequality below holds:*


$$\|\ell +\varpi^{o}{\|}^{2}\leq \|\ell {\|}^{2}+2\langle \varpi^{o},J(\ell )\rangle +2\|B\varpi^{o}{\|}^{2},\;\forall \ell ,\varpi^{o}\in E.$$

**Lemma 1.2.** *[22] Let $B_{\rho }=\{\ell \in E:\|\ell \|\leq \rho ,\rho >0\}$, where E is a UCB. Then, we can find a continuous and strictly increasing and convex function g:[0,∞)⟶[0,∞), g(0)=0 such that*


$$\|\alpha \ell +\beta \varpi^{o}+\gamma \varpi {\|}^{2}\leq \alpha \|\ell {\|}^{2}+\beta \|\varpi^{o}{\|}^{2}+\gamma \|\varpi {\|}^{2}-\alpha \beta g(\|\ell -\varpi^{o}\|),$$

*for all $\ell ,\varpi^{o},\varpi \in B_{\rho }$, and α,β,γ∈[0,1] with α+β+γ=1.*

**Lemma 1.3.** *[11] Let E be a smooth Banach space and 0/≠C⊂E be closed and convex. Let 𝐴:C⟶E be an accretive operator and $Q_{C}:E⟶C$ be a sunny non-expansive retraction from E onto C. Then, for all λ>0,*


$$S(C,𝐴)=F(Q_{C}(I-\lambda 𝐴)).$$

**Lemma 1.4.** *[21] Let ρ>0 and E be a uniformly convex. Then, we can find a convex, strictly increasing and continuous function g:[0,∞)⟶[0,∞), with g(0)=0 such that $\forall \ell ,\varpi^{o}\in B_{\rho }=\{\ell \in E:\|\ell \|\leq \rho \}$ and for any α∈[0,1], the following inequality holds:*


$$\|\alpha \ell +(1-\alpha )\varpi^{o}{\|}^{2}\leq \alpha \|\ell {\|}^{2}+(1-\alpha )\|\varpi^{o}{\|}^{2}-\alpha (1-\alpha )g\|\ell -\varpi^{o}\|.$$

**Lemma 1.5.** *[23] Let E be a real uniformly smooth Banach space, 0/≠C⊂E be closed and convex and for Ψ∈[0,∞), let ℑ:C⟶C be an Ψ-enriched non-expansive mapping with nonempty fixed point F(ℑ). If $\{\ell_{n}{\}}_{n=1}^{\infty }\subset C$ is a bounded sequence such that*

*$\lim_{n\to \infty }\|\ell -ℑ\ell \|=0$. Then, we can find a unique sunny non-expansive retraction $Q_{F(ℑ)}:C⟶F(ℑ)$ such that*


$$\underset{n\to \infty }{lim sup}\langle u-Q_{F(ℑ)}u,J(\ell_{n}-Q_{F(ℑ)}u)\rangle \leq 0,$$

*for any u∈C.*

**Lemma 1.6.** *[24] Let $\{\nu_{n}{\}}_{n=1}^{\infty }$ be a sequence in a set of nonnegative real numbers satisfying the identity*


$$\nu_{n+1}=(1-\alpha_{n})\nu_{n}+\beta_{n},\;\forall n\geq 0,$$

*where $\{\alpha_{n}{\}}_{n=0}^{\infty }$ is in (0,1) and $\{\beta_{n}{\}}_{n=0}^{\infty }$ is a sequence such that*

(*a*) $\sum_{n=1}^{\infty }\alpha_{n}=\infty$,
(*b*) ${lim sup}_{n\to \infty }\frac{\beta_{n}}{\alpha_{n}}\leq 0$ or $\sum_{n=1}^{\infty }|\beta_{n}|<\infty$.

*Then, $\lim_{n\to \infty }\nu_{n}=0$.*

The following proposition provides some fundamental properties of duality mapping:

**Proposition 1.1.** *[25] Let E be a real Banach space. For 1<q<∞, the duality $J_{q}:E⟶2^{E^{⋆}}$ has the following fundamental properties:*

*1.* $J_{q}(\ell )\neq 0\;\;\;/$* for all ℓ∈E and $D(J_{q})(:the\;dormain\;of\;J_{q})=E$;*
*2.* $J_{q}(\ell )=\|\ell {\|}^{q-1}J_{2}(\ell ),\;\forall \ell \in E(\ell \neq 0)$*;*
*3.* $J_{q}(\alpha \ell )=\alpha^{q-1}J_{q}(\ell )\;\alpha \in [0,\infty )$*;*
*4.* $J_{q}(-\ell )=-J_{q}(\ell )$*;*
*5.* *J*_*q*_ *is bounded, that is, for any bounded subset A⊂E, *J**_*q*_*(*A*) is a bounded subset in $E^{⋆}$;*
*6.* *J*_*q*_ *can be equivalently defined as the subdifferential of the functional $\psi (\ell )=q^{-1}\|\ell {\|}^{q}$, that is,*
  $$J_{q}(\ell )=\partial \psi (\ell )=\{\psi \in E^{⋆}:\psi (\varpi^{o}-\psi (\ell ))\geq \langle \psi ,\varpi^{o}-\ell \rangle ,\forall \varpi^{o}\in E\}.$$

The authors in [27–33] worked on several iterative schemes that played an important role in variational inequality problems.

## 2 Main results

In this section, the main results of the paper are proved. In the sequel, we give the following definitions.

**Definition 2.1.** Suppose that E is a real *q*-uniformly smooth Banach space for *q* > 1. A mapping Λ whose domain and range in E are denoted by D(Λ) and R(Λ),respectively, is referred to as $\left(\varkappa ,\vartheta_{i}\right)$-ESPM in the thought of Browder and Petryshyn [9] if there exist ϰ∈[0,∞), ϑ∈[0,1) and $j_{q}(\ell -\varpi^{o})\in J_{q}(\ell -\varpi^{o})$ such that for all $\ell ,\varpi^{o}\in D(\Lambda )$, the following inequality holds:

$$\langle \varkappa (\ell -\varpi^{o})+\Lambda \ell -\Lambda \varpi^{o},j_{q}((\varkappa +1)(\ell -\varpi^{o}))\rangle \leq (\varkappa +1{)}^{q}\|\ell -\varpi^{o}{\|}^{q}-\vartheta^{q-1}\|\ell -\Lambda \ell -(\varpi^{o}-\Lambda \varpi^{o}){\|}^{q}.$$ (2.1)

**Remark 2.1.** Some immediate consequences of (2.1) are:

1. If *q* = 2, then (2.1) reduces to
  $$\langle \varkappa (\ell -\varpi^{o})+\Lambda \ell -\Lambda \varpi^{o},j_{2}((\varkappa +1)(\ell -\varpi^{o}))\rangle \leq (\varkappa +1{)}^{2}\|\ell -\varpi^{o}{\|}^{2}-\vartheta \|\ell -\Lambda \ell -(\varpi^{o}-\Lambda \varpi^{o}){\|}^{2},$$
  a notion studied in [2,3].
2. If ϰ=0, then (2.1) reduces to
  $$\langle \Lambda \ell -\Lambda \varpi^{o},j_{q}(\ell -\varpi^{o})\rangle \leq \|\ell -\varpi^{o}{\|}^{q}-\vartheta^{q-1}\|\ell -\Lambda \ell -(\varpi^{o}-\Lambda \varpi^{o}){\|}^{q},$$
  a notion examined in [32].
3. If E is a real Hilbert space, then (2.1) reduces to
  $$\langle \varkappa (\ell -\varpi^{o})+\Lambda \ell -\Lambda \varpi^{o},(\varkappa +1)(\ell -\varpi^{o})\rangle \leq (\varkappa +1{)}^{2}\|\ell -\varpi^{o}{\|}^{2}-\vartheta \|\ell -\Lambda \ell -(\varpi^{o}-\Lambda \varpi^{o}){\|}^{2},$$
  a notion investigated in [2].
4. If ϰ=0 and q=2, then (2.1) reduces to
  $$\langle \Lambda \ell -\Lambda \varpi^{o},j(\ell -\varpi^{o})\rangle \leq \|\ell -\varpi^{o}{\|}^{2}-\vartheta \|\ell -\Lambda \ell -(\varpi^{o}-\Lambda \varpi^{o}){\|}^{2},$$
  a notion considered in [6,30].
5. If q=2,ϑ=0, then (2.1) reduces to
  $$\langle \varkappa (\ell -\varpi^{o})+\Lambda \ell -\Lambda \varpi^{o},j((\varkappa +1)(\ell -\varpi^{o}))\rangle \leq (\varkappa +1{)}^{2}\|\ell -\varpi^{o}{\|}^{2},$$
  a notion studied in [4,5].

Set $\gamma =\frac{1}{\varkappa +1}$. Then, γ∈(0,1]. Consequently, (2.1) becomes


$$\langle \frac{\left(1-\gamma \right)}{\gamma }(\ell -\varpi^{o})+\;\Lambda \ell -\Lambda \varpi^{o},j_{q}(\frac{1}{\gamma }(\ell -\varpi^{o}))\rangle \leq \frac{1}{\gamma^{q}}\|\ell -\varpi^{o}{\|}^{q}-\vartheta^{q-1}\|\ell -\Lambda \ell -(\varpi^{o}-\Lambda \varpi^{o}){\|}^{q},$$

which, by Proposition 1.1 (3) and on simplifying, yields

$$\langle \Lambda_{\gamma }\ell -\Lambda_{\gamma }\varpi^{o},j_{q}(\ell -\varpi^{o})\rangle \leq \|\ell -\varpi^{o}{\|}^{q}-\vartheta^{q-1}\|\ell -\Lambda_{\gamma }\ell -(\varpi^{o}-\Lambda_{\gamma }\varpi^{o}){\|}^{q},$$ (2.2)

where $\Lambda_{\gamma }=(1-\gamma )I+\gamma \Lambda$, *I* is the identity mapping on E. It is not hard to see that inequality (2.2) is equivalent to

$$\langle (I-\Lambda_{\gamma })\ell -(I-\Lambda_{\gamma })\varpi^{o},j_{q}(\ell -\varpi^{o})\rangle \geq \vartheta^{q-1}\|\ell -\Lambda_{\gamma }\ell -(\varpi^{o}-\Lambda_{\gamma }\varpi^{o}){\|}^{q},$$ (2.3)

Observe that the average operator $\Lambda_{\gamma }$ in both (2.2) and (2.3) is ϑ−strictly pseudocontractive.

**Definition 2.2.** Let E be a real Banach space, 0/≠K⊂E and $\{{ℑ}_{r}{\}}_{i=1}^{m},\{{ℑ}_{r}{\}}_{i=1}^{m}:K⟶K$ be finite families of mappings. For each r=1,2,⋯,N, let $\delta_{i}=(\delta_{1}^{r},\delta_{2}^{r},\delta_{3}^{r})\in J\times J\times J$, where J=[0,1] and $\delta_{1}^{r}+\delta_{2}^{r}+\delta_{3}^{r}=1$. The mapping $D^{A}:C⟶C$ is defined as follows:


$$G_{0}={ℑ}_{\omega ,1}=I$$

$$G_{1}={ℑ}_{\omega ,1}(\delta_{1}^{1}{ℑ}_{\omega ,1}G_{0}+\delta_{2}^{1}G_{0}+\delta_{3}^{1}I)$$

$$G_{2}={ℑ}_{\omega ,2}(\delta_{1}^{2}{ℑ}_{\omega ,2}G_{1}+\delta_{2}^{2}G_{1}+\delta_{3}^{2}I)$$

$$G_{3}={ℑ}_{\omega ,3}(\delta_{1}^{3}{ℑ}_{\omega ,3}G_{2}+\delta_{2}^{3}G_{2}+\delta_{3}^{3}I)$$

⋮

$$G_{m-1}={ℑ}_{\omega ,m-1}(\delta_{1}^{m-1}{ℑ}_{\omega ,m-1}G_{m-2}+\delta_{2}^{m-1}G_{m-2}+\delta_{3}^{m-1}I)$$

$$D=G_{m}={ℑ}_{\omega ,m}(\delta_{1}^{m}{ℑ}_{\omega ,N}G_{m-1}+\delta_{2}^{m}G_{m-1}+\delta_{3}^{m}I),$$

where $m\in N,{ℑ}_{\omega ,r}=(1-\omega )I+\omega {ℑ}_{r}$ and ${ℑ}_{\omega ,r}=(1-\omega )I+\omega {ℑ}_{r}$, i=1,2,…,m. This mapping is known as Ψ-enriched ${\text{D}}^{\text{A}}$-mapping developed by ${ℑ}_{\omega ,1},{ℑ}_{\omega ,2},\cdots ,{ℑ}_{\omega ,m},{ℑ}_{\omega ,1},{ℑ}_{\omega ,2},\cdots ,{ℑ}_{\omega ,m}$ and $\delta_{1}^{r},\delta_{2}^{r},\delta_{3}^{r},\;r=1,2,\ldots ,m.$

**Remark 2.2.** If Ψ=0, then ω=1 and Ψ-enriched ${\text{D}}^{\text{A}}$-mapping reduces to ${\text{S}}^{\text{A}}$-mapping. Thus, the class of Ψ-enriched ${\text{D}}^{\text{A}}$-mapping properly includes the classes of *S*-mappings, ${\text{S}}^{\text{A}}$-mappings and ${\text{D}}^{\text{A}}$-mappings.

In the course of proving our main results, the following assumptions are used.

**Assumption $\Delta_{1}$**

1. E is a 2-uniformly smooth and uniformly convex Banach space and 0/≠C⊂E is closed and convex;
2. $\{{ℑ}_{r}{\}}_{i=1}^{m}:C⟶C$ is a finite family of $\left(\Psi ,\lambda_{r}\right)$-enriched strictly pseudocontractive mappings (*ESPM*) and $\{{ℑ}_{i}{\}}_{r=1}^{m}:C⟶C$ is a finite family of $\Psi_{i}$-enriched nonexpansive mappings (*ENM*) with ${⋂}_{r=1}^{m}F({ℑ}_{i})\cap {⋂}_{r=1}^{m}F({ℑ}_{r})\neq 0\;\;\;/$ and $\lambda =\max\{\lambda_{1},\lambda_{2},\cdots ,\lambda_{m}\}$ with $B^{2}\leq \lambda$, where *B* is the 2-uniformly smooth constant of E;
3. $\delta_{r}=(\delta_{1}^{i},\delta_{2}^{r},\delta_{3}^{r})\in J\times J\times J$, where J=[0,1], $\delta_{1}^{r}+\delta_{2}^{r}+\delta_{3}^{r}=1,\delta_{1}^{r},\delta_{2}^{i}\in (0,1]$ and $\delta_{3}^{i}\in (0,1)$ for all r=1,2,⋯,m;
4. ${\text{D}}^{\text{A}}$ be an Ψ-enriched ${\text{D}}^{\text{A}}$-mapping developed by the sequences $\{{ℑ}_{\omega ,i}{\}}_{r=1}^{m},\{{ℑ}_{\omega ,r}{\}}_{r=1}^{m}$ and $\{\delta_{r}{\}}_{r=1}^{m}$;
5. A,B are ξ- and *ρ*-inverse strongly accretive mappings of C onto E, respectively; $Q_{C}$ is a sunny non-expansive retraction from E onto C.

**Assumption $\Delta_{2}$**

$\{\pi_{n}{\}}_{n=1}^{\infty },\{\omega_{n}{\}}_{n=1}^{\infty },\{\varrho_{n}{\}}_{n=1}^{\infty },\{\xi_{n}{\}}_{n=1}^{\infty }$ and $\{\varphi_{n}{\}}_{n=1}^{\infty }$ are sequences in [0,1] with $\pi_{n}+\omega_{n}+\varrho_{n}+\varphi_{n}+\xi_{n}=1$ and satisfying the conditions below:

(*a*) $\underset{n\to \infty }{\lim}\pi_{n}=0,\;\sum_{n=1}^{\infty }\pi_{n}=\infty$;
(*b*) $\left\{\varrho_{n}{\}}_{n=1}^{\infty },\{\varphi_{n}{\}}_{n=1}^{\infty },\{\xi_{n}{\}}_{n=1}^{\infty }\subseteq [c,d]\subset (0,1\right)$, for some c,d>0,∀n≥1;
(*c*) $\underset{n=1}{\overset{\infty }{\sum }}|\pi_{n+1}\;-\;\pi_{n}|<\infty ,\;\underset{n=1}{\overset{\infty }{\sum }}|\omega_{n+1}\;-\;\omega_{n}|<\infty ,\;\underset{n=1}{\overset{\infty }{\sum }}|\rho_{n+1}\;-\;\rho_{n}|<\infty ,\underset{n=1}{\overset{\infty }{\sum }}|\varphi_{n+1}\;-\;\varphi_{n}|<\infty ,\;\underset{n=1}{\overset{\infty }{\sum }}|\xi_{n+1}\;-\;\xi_{n}|<\infty$;
(*d*) $0<\underset{n\to \infty }{lim inf}\omega_{n}\leq \underset{n\to \infty }{lim sup}\omega_{n}<1$;
(*e*) $a\in (0,\frac{\xi }{B^{2}})$ and $b\in (0,\frac{\varkappa }{B^{2}})$.

Now, we prove the following result by using Ψ-enriched ${\text{D}}^{\text{A}}$-mapping in a 2-USB and UCB.

**Theorem 2.1.** *Let $E,\{{ℑ}_{i}{\}}_{i=1}^{m},\{{ℑ}_{i}{\}}_{i=1}^{m},\delta_{i}$ and ${\text{D}}^{\text{A}}$ be as described in Assumption $\Delta_{1}$. Then, $F(D^{A})={⋂}_{i=1}^{m}F({ℑ}_{i})\cap {⋂}_{i=1}^{m}F({ℑ}_{i})$ and Ψ-enriched ${\text{D}}^{\text{A}}$-mapping is nonexpansive.*

*Proof:* Let m∈N, $\ell_{0}\in F(D^{A})$ and $\ell^{⋆}\in {⋂}_{r=1}^{m}F({ℑ}_{r})\cap {⋂}_{r=1}^{m}F({ℑ}_{r})$. Then, we have the following estimates: Set $Q=\delta_{1}^{m}{ℑ}_{\omega ,m}G_{m-1}\ell_{0}+\delta_{2}^{m}G_{m-1}\ell_{0}+\delta_{3}^{m}\ell_{0}$. Since


$$Q=\delta_{1}^{m}((1-\omega )I+\omega {ℑ}_{m})G_{m-1}\ell_{0}+\delta_{2}^{m}G_{m-1}\ell_{0}+\delta_{3}^{m}\ell_{0}$$

$$=\delta_{1}^{m}((1-\frac{1}{\Psi +1})G_{m-1}\ell_{0}+\frac{1}{\Psi +1}{ℑ}_{m}G_{m-1}\ell_{0})+\delta_{2}^{m}G_{m-1}\ell_{0}+\delta_{3}^{m}\ell_{0}$$

$$=\delta_{1}^{m}(\frac{\Psi }{\Psi +1}G_{m-1}\ell_{0}+\frac{1}{\Psi +1}{ℑ}_{m}G_{m-1}\ell_{0})+\delta_{2}^{m}G_{m-1}\ell_{0}+\delta_{3}^{m}\ell_{0}$$

$$=\frac{\delta_{1}^{m}}{\Psi +1}(\Psi G_{N-1}\ell_{0}+{ℑ}_{m}G_{m-1}\ell_{0})+\delta_{2}^{m}G_{m-1}\ell_{0}+\delta_{3}^{m}\ell_{0}$$

and ${ℑ}_{\omega ,m}=(1-\omega )I+\omega {ℑ}_{m}$, it follows that


$$\|\ell_{0}-\ell^{⋆}{\|}^{2}=\|{ℑ}_{\omega ,m}(\delta_{1}^{m}{ℑ}_{\omega ,m}G_{m-1}\ell_{0}+\delta_{2}^{m}G_{m-1}\ell_{0}+\delta_{3}^{m}\ell_{0})-\ell^{⋆}{\|}^{2}$$

$$=\|\frac{\Psi }{\Psi +1}(\frac{\delta_{1}^{m}}{\Psi +1}(\Psi G_{m-1}\ell_{0}+{ℑ}_{m}G_{m-1}\ell_{0})+\delta_{2}^{m}G_{m-1}\ell_{0}+\delta_{3}^{m}\ell_{0})\;+\frac{1}{\Psi +1}{ℑ}_{m}(\frac{\delta_{1}^{m}}{\Psi +1}(\Psi G_{m-1}\ell_{0}+{ℑ}_{m}G_{m-1}\ell_{0})+\delta_{2}^{m}G_{m-1}\ell_{0}+\delta_{3}^{m}\ell_{0})-\ell^{⋆}{\|}^{2}$$

$$=\frac{1}{(\Psi +1{)}^{2}}\|\Psi ((\frac{\delta_{1}^{m}}{\Psi +1}(\Psi G_{m-1}\ell_{0}+{ℑ}_{m}G_{m-1}\ell_{0}))+\delta_{2}^{m}G_{m-1}\ell_{0}+\delta_{3}^{m}\ell_{0}\;\;-(\frac{\delta_{1}^{m}}{\Psi +1}(\Psi G_{m-1}\ell^{⋆}+{ℑ}_{m}G_{m-1}\ell^{⋆})+\delta_{2}^{m}G_{m-1}\ell^{⋆}+\delta_{3}^{m}\ell^{⋆}))\;\;+{ℑ}_{m}(\frac{\delta_{1}^{m}}{\Psi +1}(\Psi G_{m-1}\ell_{0}+{ℑ}_{m}G_{m-1}\ell_{0})+\delta_{2}^{m}G_{m-1}\ell_{0}+\delta_{3}^{m}\ell_{0})\;\;-{ℑ}_{m}(\frac{\delta_{1}^{m}}{\Psi +1}(\Psi G_{m-1}\ell^{⋆}+{ℑ}_{m}G_{m-1}\ell^{⋆})+\delta_{2}^{m}G_{m-1}\ell^{⋆}+\delta_{3}^{m}\ell^{⋆}){\|}^{2}$$

$$\leq \frac{1}{(\Psi +1{)}^{2}}[(\Psi \;+\;1{)}^{2}\|\frac{\delta_{1}^{m}}{\Psi \;+\;1}(\Psi G_{m-1}\ell_{0}\;+\;{ℑ}_{N}G_{m-1}\ell_{0})\;+\;\delta_{2}^{m}G_{m-1}\ell_{0}\;+\;\delta_{3}^{m}\ell_{0}\;-\;\ell^{⋆}{\|}^{2}]$$

$$=\|(1-\delta_{3}^{m})[\frac{\delta_{1}^{m}}{\left(\Psi +1)(1-\delta_{3}^{m}\right)}(\Psi G_{m-1}\ell_{0}+{ℑ}_{m}G_{m-1}\ell_{0})+\frac{\delta_{2}^{m}}{\left(1-\delta_{3}^{m}\right)}G_{m-1}\ell_{0}]\;\;+\delta_{3}^{m}\ell_{0}-\ell^{⋆}{\|}^{2}$$

$$=\|(1-\delta_{3}^{m})[\frac{\delta_{1}^{m}}{\left(\Psi +1)(1-\delta_{3}^{m}\right)}(\Psi (G_{m-1}\ell_{0}-\ell^{⋆})+{ℑ}_{m}G_{m-1}\ell_{0}-\ell^{⋆})\;\;+\frac{\delta_{2}^{m}}{\left(1-\delta_{3}^{m}\right)}(G_{m-1}\ell_{0}-\ell^{⋆})]+\delta_{3}^{m}(\ell_{0}-\ell^{⋆}){\|}^{2}$$

$$\leq \frac{\left(1-\delta_{3}^{m}\right)}{(\Psi +1{)}^{2}}\|\frac{\delta_{1}^{m}}{\left(1-\delta_{3}^{m}\right)}(\Psi (G_{m-1}\ell_{0}-\ell^{⋆})+{ℑ}_{m}G_{m-1}\ell_{0}-\ell^{⋆})\;\;+\frac{\delta_{2}^{m}}{\left(1-\delta_{3}^{m}\right)}((\Psi +1)(G_{m-1}\ell_{0}-\ell^{⋆})){\|}^{2}+\delta_{3}^{m}\|\ell_{0}-\ell^{⋆}{\|}^{2}$$

$$=\frac{\left(1-\delta_{3}^{m}\right)}{(\Psi +1{)}^{2}}\|\frac{\delta_{1}^{m}}{\left(1-\delta_{3}^{m}\right)}(\Psi (G_{m-1}\ell_{0}-\ell^{⋆})+{ℑ}_{m}G_{m-1}\ell_{0}-\ell^{⋆})\;\;+(1-\frac{\delta_{1}^{m}}{1-\delta_{3}^{m}})((\Psi +1)(G_{m-1}\ell_{0}-\ell^{⋆})){\|}^{2}+\delta_{3}^{m}\|\ell_{0}-\ell^{⋆}{\|}^{2}$$

$$=\frac{\left(1-\delta_{3}^{m}\right)}{(\Psi +1{)}^{2}}\|\frac{\delta_{1}^{m}}{\left(1-\delta_{3}^{m}\right)}[(\Psi (G_{m-1}\ell_{0}-\ell^{⋆})+{ℑ}_{m}G_{m-1}\ell_{0}-\ell^{⋆})-(\Psi +1)(G_{m-1}\ell_{0}-\ell^{⋆})]\;\;+(\Psi +1)(G_{m-1}\ell_{0}-\ell^{⋆}){\|}^{2}+\delta_{3}^{m}\|\ell_{0}-\ell^{⋆}{\|}^{2}$$

$$\leq \frac{\left(1-\delta_{3}^{m}\right)}{(\Psi +1{)}^{2}}\{(\Psi +1{)}^{2}\|G_{m-1}\ell_{0}-\ell^{⋆}{\|}^{2}+2\frac{\delta_{1}^{m}}{1-\delta_{3}^{m}}\langle (\Psi (G_{m-1}\ell_{0}-\ell^{⋆})+{ℑ}_{m}G_{m-1}\ell_{0}-\ell^{⋆})\;\;-(\Psi +1)(G_{m-1}\ell_{0}-\ell^{⋆}),j((\Psi +1)(G_{m-1}\ell_{0}-\ell^{⋆}))\rangle \;\;+2B^{2}(\frac{\delta_{1}^{m}}{1-\delta_{3}^{m}}{)}^{2}\|(\Psi (G_{m-1}\ell_{0}-\ell^{⋆})+{ℑ}_{m}G_{m-1}\ell_{0}-\ell^{⋆})-(\Psi +1)(G_{m-1}\ell_{0}-\ell^{⋆}){\|}^{2}\}\;\;+\delta_{3}^{m}\|\ell_{0}-\ell^{⋆}{\|}^{2}$$

$$=\frac{\left(1-\delta_{3}^{m}\right)}{(\Psi +1{)}^{2}}\{(\Psi +1{)}^{2}\|G_{m-1}\ell_{0}-\ell^{⋆}{\|}^{2}+2\frac{\delta_{1}^{m}}{1-\delta_{3}^{m}}\langle (\Psi (G_{m-1}\ell_{0}-\ell^{⋆})\;\;+{ℑ}_{m}G_{m-1}\ell_{0}-\ell^{⋆}),j((\Psi +1)(G_{m-1}\ell_{0}-\ell^{⋆}))\rangle \;\;+2\frac{\delta_{1}^{m}(\Psi +1{)}^{2}}{1-\delta_{3}^{m}}\langle \ell^{⋆}-G_{m-1}\ell_{0},j(G_{m-1}\ell_{0}-\ell^{⋆})\rangle \;\;+2B^{2}(\frac{\delta_{1}^{m}}{1-\delta_{3}^{m}}{)}^{2}\|{ℑ}_{m}G_{m-1}\ell_{0}-G_{m-1}\ell_{0}{\|}^{2}\}+\delta_{3}^{m}\|\ell_{0}-\ell^{⋆}{\|}^{2}$$

$$\leq \frac{\left(1-\delta_{3}^{m}\right)}{(\Psi +1{)}^{2}}\{(\Psi +1{)}^{2}\|G_{m-1}\ell_{0}-\ell^{⋆}{\|}^{2}+2\frac{\delta_{1}^{m}}{1-\delta_{3}^{m}}[(\Psi +1{)}^{2}\|G_{m-1}\ell_{0}-\ell^{⋆}{\|}^{2}\;\;-\vartheta \|(I-{ℑ}_{m})G_{m-1}\ell_{0}{\|}^{2}]-2\frac{\delta_{1}^{m}(\Psi +1{)}^{2}}{1-\delta_{3}^{m}}\|\ell^{⋆}-G_{m-1}\ell_{0}{\|}^{2}\;\;+2B^{2}(\frac{\delta_{1}^{m}}{1-\delta_{3}^{m}}{)}^{2}\|{ℑ}_{N}G_{m-1}\ell_{0}-G_{m-1}\ell_{0}{\|}^{2}\}+\delta_{3}^{m}\|\ell_{0}-\ell^{⋆}{\|}^{2}$$

$$=\frac{\left(1-\delta_{3}^{m}\right)}{(\Psi +1{)}^{2}}\{(\Psi +1{)}^{2}\|G_{m-1}\ell_{0}-\ell^{⋆}{\|}^{2}-2\frac{\delta_{1}^{m}}{1-\delta_{3}^{m}}(\vartheta -B^{2}(\frac{\delta_{1}^{m}}{1-\delta_{3}^{m}}))\|(I-{ℑ}_{m})G_{m-1}\ell_{0}{\|}^{2}\}\;\;+\delta_{3}^{m}\|\ell_{0}-\ell^{⋆}{\|}^{2}$$

$$\leq (1-\delta_{3}^{m})\|G_{m-1}\ell_{0}-\ell^{⋆}{\|}^{2}+\delta_{3}^{m}\|\ell_{0}-\ell^{⋆}{\|}^{2}$$

$$\leq (1-\delta_{3}^{m})[(1-\delta_{3}^{m-1})\|G_{m-2}\ell_{0}-\ell^{⋆}{\|}^{2}+\delta_{3}^{m-1}\|\ell_{0}-\ell^{⋆}{\|}^{2}]+\delta_{3}^{N}\|\ell_{0}-\ell^{⋆}{\|}^{2}$$

$$=(1-\delta_{3}^{m-1})(1-\delta_{3}^{m})\|G_{m-2}\ell_{0}-\ell^{⋆}{\|}^{2}+(1-(1-\delta_{3}^{m-1})(1-\delta_{3}^{m}))\|\ell_{0}-\ell^{⋆}{\|}^{2}$$

$$=\prod_{r=m-1}^{m}(1-\delta_{3}^{r})\|G_{m-2}\ell_{0}-\ell^{⋆}{\|}^{2}+(1-\prod_{r=m-1}^{m}(1-\delta_{3}^{r}))\|\ell_{0}-\ell^{⋆}{\|}^{2}$$

$$\leq \prod_{r=3}^{m}(1-\delta_{3}^{i})\|G_{2}\ell_{0}-\ell^{⋆}{\|}^{2}+(1-\prod_{r=3}^{m}(1-\delta_{3}^{r}))\|\ell_{0}-\ell^{⋆}{\|}^{2}$$

$$=\prod_{r=3}^{m}(1-\delta_{3}^{r})\|{ℑ}_{\omega ,2}(\delta_{1}^{2}{ℑ}_{\omega ,2}G_{1}+\delta_{2}^{2}G_{1}+\delta_{3}^{2}I)\ell_{0}-\ell^{⋆}{\|}^{2}+(1-\prod_{r=3}^{m}(1-\delta_{3}^{r}))\|\ell_{0}-\ell^{⋆}{\|}^{2}$$

$$=\prod_{r=3}^{m}(1-\delta_{3}^{r})\|\frac{\Psi }{\Psi +1}(\frac{\delta_{1}^{2}}{\Psi +1}(\Psi G_{1}\ell_{0}+{ℑ}_{2}G_{1}\ell_{0})+\delta_{2}^{2}G_{1}\ell_{0}+\delta_{3}^{2}\ell_{0})\;\;+\frac{1}{\Psi +1}{ℑ}_{2}(\frac{\delta_{1}^{2}}{\Psi +1}(\Psi G_{1}\ell_{0}+{ℑ}_{2}G_{1}\ell_{0})+\delta_{2}^{2}G_{1}\ell_{0}+\delta_{3}^{2}\ell_{0})-\ell^{⋆}{\|}^{2}\;\;+(1-\prod_{r=3}^{m}(1-\delta_{3}^{r}))\|\ell_{0}-\ell^{⋆}{\|}^{2}$$

$$=\prod_{r=3}^{m}(1-\delta_{3}^{r})\{\frac{1}{(\Psi +1{)}^{2}}\|\Psi ((\frac{\delta_{1}^{2}}{\Psi +1}(\Psi G_{1}\ell_{0}+{ℑ}_{2}G_{1}\ell_{0}))+\delta_{2}^{2}G_{1}\ell_{0}+\delta_{3}^{2}\ell_{0}\;\;-(\frac{\delta_{1}^{2}}{\Psi +1}(\Psi G_{1}\ell^{⋆}+{ℑ}_{2}G_{N-1}\ell^{⋆})+\delta_{2}^{2}G_{1}\ell^{⋆}+\delta_{3}^{2}\ell^{⋆}))\;\;+{ℑ}_{2}(\frac{\delta_{1}^{2}}{\Psi +1}(\Psi G_{1}\ell_{0}+{ℑ}_{2}G_{1}\ell_{0})+\delta_{2}^{2}G_{1}\ell_{0}+\delta_{3}^{2}\ell_{0})\;\;-{ℑ}_{2}(\frac{\delta_{1}^{2}}{\Psi +1}(\Psi G_{1}\ell^{⋆}+{ℑ}_{2}G_{1}\ell^{⋆})+\delta_{2}^{2}G_{1}\ell^{⋆}+\delta_{3}^{2}\ell^{⋆}){\|}^{2}\}+(1-\prod_{r=3}^{m}(1-\delta_{3}^{r}))\|\ell_{0}-\ell^{⋆}{\|}^{2}$$

$$\leq \prod_{r=3}^{m}(1-\delta_{3}^{r})\{\frac{1}{(\Psi +1{)}^{2}}[(\Psi +1{)}^{2}\|\frac{\delta_{1}^{2}}{\Psi +1}(\Psi G_{1}\ell_{0}+{ℑ}_{2}G_{1}\ell_{0})+\delta_{2}^{2}G_{1}\ell_{0}+\delta_{3}^{2}\ell_{0}-\ell^{⋆}{\|}^{2}]\}\;\;+(1-\prod_{r=3}^{m}(1-\delta_{3}^{i}))\|\ell_{0}-\ell^{⋆}{\|}^{2}$$

$$=\prod_{r=3}^{m}(1-\delta_{3}^{r})\{\|(1-\delta_{3}^{2})[\frac{\delta_{1}^{2}}{\left(\Psi +1)(1-\delta_{3}^{2}\right)}(\Psi G_{1}\ell_{0}+{ℑ}_{2}G_{1}\ell_{0})+\frac{\delta_{2}^{2}}{\left(1-\delta_{3}^{2}\right)}G_{1}\ell_{0}]+\delta_{3}^{2}\ell_{0}-\ell^{⋆}{\|}^{2}\}\;\;+(1-\prod_{r=3}^{m}(1-\delta_{3}^{r}))\|\ell_{0}-\ell^{⋆}{\|}^{2}$$

$$=\prod_{r=3}^{m}(1-\delta_{3}^{r})\|(1-\delta_{3}^{2})[\frac{\delta_{1}^{2}}{\left(\Psi +1)(1-\delta_{3}^{2}\right)}(\Psi (G_{1}\ell_{0}-\ell^{⋆})+{ℑ}_{2}G_{1}\ell_{0}-\ell^{⋆})\;\;+\frac{\delta_{2}^{2}}{\left(1-\delta_{3}^{2}\right)}(G_{1}\ell_{0}-\ell^{⋆})]+\delta_{3}^{2}(\ell_{0}-\ell^{⋆}){\|}^{2}+(1-\prod_{r=3}^{m}(1-\delta_{3}^{r}))\|\ell_{0}-\ell^{⋆}{\|}^{2}$$

$$\leq \prod_{r=3}^{m}(1-\delta_{3}^{r})\{\frac{\left(1-\delta_{3}^{2}\right)}{(\Psi +1{)}^{2}}\|\frac{\delta_{1}^{2}}{\left(1-\delta_{3}^{2}\right)}(\Psi (G_{1}\ell_{0}-\ell^{⋆})+{ℑ}_{2}G_{1}\ell_{0}-\ell^{⋆})\;\;+\frac{\delta_{2}^{2}}{\left(1-\delta_{3}^{2}\right)}((\Psi +1)(G_{1}\ell_{0}-\ell^{⋆})){\|}^{2}+\delta_{3}^{2}\|\ell_{0}-\ell^{⋆}{\|}^{2}\}+(1-\prod_{r=3}^{m}(1-\delta_{3}^{i}))\|\ell_{0}-\ell^{⋆}{\|}^{2}$$

$$=\prod_{r=3}^{m}(1-\delta_{3}^{r})\{\frac{\left(1-\delta_{3}^{2}\right)}{(\Psi +1{)}^{2}}\|\frac{\delta_{1}^{2}}{\left(1-\delta_{3}^{2}\right)}(\Psi (G_{1}\ell_{0}-\ell^{⋆})+{ℑ}_{2}G_{1}\ell_{0}-\ell^{⋆})\;\;+(1-\frac{\delta_{1}^{2}}{1-\delta_{3}^{2}})((\Psi +1)(G_{1}\ell_{0}-\ell^{⋆})){\|}^{2}+\delta_{3}^{2}\|\ell_{0}-\ell^{⋆}{\|}^{2}\}+(1-\prod_{r=3}^{m}(1-\delta_{3}^{r}))\|\ell_{0}-\ell^{⋆}{\|}^{2}$$

$$=\prod_{r=3}^{m}(1-\delta_{3}^{r})\{\frac{\left(1-\delta_{3}^{2}\right)}{(\Psi +1{)}^{2}}\|\frac{\delta_{1}^{2}}{\left(1-\delta_{3}^{2}\right)}[(\Psi (G_{1}\ell_{0}-\ell^{⋆})+{ℑ}_{2}G_{1}\ell_{0}-\ell^{⋆})-(\Psi +1)(G_{1}\ell_{0}-\ell^{⋆})]\;\;+(\Psi +1)(G_{1}\ell_{0}-\ell^{⋆}){\|}^{2}+\delta_{3}^{2}\|\ell_{0}-\ell^{⋆}{\|}^{2}\}+(1-\prod_{r=3}^{m}(1-\delta_{3}^{r}))\|\ell_{0}-\ell^{⋆}{\|}^{2}$$ (2.4)

$$\leq \prod_{r=3}^{m}(1-\delta_{3}^{r})\{\frac{\left(1-\delta_{3}^{2}\right)}{(\Psi +1{)}^{2}}\{(\Psi +1{)}^{2}\|G_{1}\ell_{0}-\ell^{⋆}{\|}^{2}+2\frac{\delta_{1}^{2}}{1-\delta_{3}^{2}}\langle (\Psi (G_{1}\ell_{0}-\ell^{⋆})+{ℑ}_{2}G_{1}\ell_{0}-\ell^{⋆})\;\;-(\Psi +1)(G_{1}\ell_{0}-\ell^{⋆}),j((\Psi +1)(G_{1}\ell_{0}-\ell^{⋆}))\rangle \;\;+2B^{2}(\frac{\delta_{1}^{2}}{1-\delta_{3}^{2}}{)}^{2}\|(\Psi (G_{1}\ell_{0}-\ell^{⋆})+{ℑ}_{2}G_{1}\ell_{0}-\ell^{⋆})-(\Psi +1)(G_{1}\ell_{0}-\ell^{⋆}){\|}^{2}\}\;\;+\delta_{3}^{2}\|\ell_{0}-\ell^{⋆}{\|}^{2}\}+(1-\prod_{r=3}^{m}(1-\delta_{3}^{r}))\|\ell_{0}-\ell^{⋆}{\|}^{2}$$

$$=\prod_{r=3}^{m}(1-\delta_{3}^{r})\{\frac{\left(1-\delta_{3}^{2}\right)}{(\Psi +1{)}^{2}}\{(\Psi +1{)}^{2}\|G_{1}\ell_{0}-\ell^{⋆}{\|}^{2}+2\frac{\delta_{1}^{2}}{1-\delta_{3}^{2}}\langle \Psi (G_{1}\ell_{0}-\ell^{⋆})\;\;+{ℑ}_{2}G_{1}\ell_{0}-\ell^{⋆},j((\Psi +1)(G_{1}\ell_{0}-\ell^{⋆}))\rangle \;\;+2\frac{\delta_{1}^{2}(\Psi +1{)}^{2}}{1-\delta_{3}^{2}}\langle \ell^{⋆}-G_{1}\ell_{0},j(G_{1}\ell_{0}-\ell^{⋆})\rangle \;\;+2B^{2}(\frac{\delta_{1}^{2}}{1-\delta_{3}^{2}}{)}^{2}\|{ℑ}_{2}G_{1}\ell_{0}-G_{1}\ell_{0}{\|}^{2}\}+\delta_{3}^{2}\|\ell_{0}-\ell^{⋆}{\|}^{2}\}+(1-\prod_{r=3}^{m}(1-\delta_{3}^{r}))\|\ell_{0}-\ell^{⋆}{\|}^{2}$$

$$\leq \prod_{r=3}^{m}(1-\delta_{3}^{r})\{\frac{\left(1-\delta_{3}^{2}\right)}{(\Psi +1{)}^{2}}\{(\Psi +1{)}^{2}\|G_{1}\ell_{0}-\ell^{⋆}{\|}^{2}+2\frac{\delta_{1}^{2}}{1-\delta_{3}^{2}}[(\Psi +1{)}^{2}\|G_{1}\ell_{0}-\ell^{⋆}{\|}^{2}\;\;-\lambda \|(I-{ℑ}_{2})G_{1}\ell_{0}{\|}^{2}]-2\frac{\delta_{1}^{2}(\Psi +1{)}^{2}}{1-\delta_{3}^{2}}\|\ell^{⋆}-G_{1}\ell_{0}{\|}^{2}\;\;+2B^{2}(\frac{\delta_{1}^{2}}{1-\delta_{3}^{2}}{)}^{2}\|{ℑ}_{2}G_{1}\ell_{0}-G_{1}\ell_{0}{\|}^{2}+\delta_{3}^{2}\|\ell_{0}-\ell^{⋆}{\|}^{2}\}\}+(1-\prod_{r=3}^{m}(1-\delta_{3}^{r}))\|\ell_{0}-\ell^{⋆}{\|}^{2}$$

$$=\prod_{r=3}^{m}(1-\delta_{3}^{r})\{\frac{\left(1-\delta_{3}^{2}\right)}{(\Psi +1{)}^{2}}\{(\Psi +1{)}^{2}\|G_{1}\ell_{0}-\ell^{⋆}{\|}^{2}\;\;-2\frac{\delta_{1}^{2}}{1-\delta_{3}^{2}}(\lambda -B^{2}(\frac{\delta_{1}^{2}}{1-\delta_{3}^{2}}))\|(I-{ℑ}_{2})G_{1}\ell_{0}{\|}^{2}\}\;\;+\delta_{3}^{2}\|\ell_{0}-\ell^{⋆}{\|}^{2}\}+(1-\prod_{r=3}^{m}(1-\delta_{3}^{r}))\|\ell_{0}-\ell^{⋆}{\|}^{2}$$

$$\leq \prod_{r=3}^{m}(1-\delta_{3}^{r})[(1-\delta_{3}^{2})\|G_{1}\ell_{0}-\ell^{⋆}{\|}^{2}+\delta_{3}^{2}\|\ell_{0}-\ell^{⋆}{\|}^{2}]+(1-\prod_{r=3}^{m}(1-\delta_{3}^{r}))\|\ell_{0}-\ell^{⋆}{\|}^{2}$$

$$=\prod_{r=2}^{m}(1-\delta_{3}^{r})\|G_{1}\ell_{0}-\ell^{⋆}{\|}^{2}+(1-\prod_{r=2}^{m}(1-\delta_{3}^{r}))\|\ell_{0}-\ell^{⋆}{\|}^{2}$$

$$=\prod_{r=3}^{m}(1-\delta_{3}^{r})\|{ℑ}_{\omega ,1}(\delta_{1}^{1}{ℑ}_{\omega ,1}G_{0}+\delta_{2}^{1}G_{0}+\delta_{3}^{1}I)\ell_{0}-\ell^{⋆}{\|}^{2}+(1-\prod_{r=3}^{m}(1-\delta_{3}^{r}))\|\ell_{0}-\ell^{⋆}{\|}^{2}$$

$$=\prod_{r=3}^{m}(1-\delta_{3}^{r})\|\frac{\Psi }{\Psi +1}(\frac{\delta_{1}^{1}}{\Psi +1}(\Psi G_{0}\ell_{0}+{ℑ}_{1}G_{0}\ell_{0})+\delta_{2}^{1}G_{1}\ell_{0}+\delta_{3}^{1}\ell_{0})\;\;+\frac{1}{\Psi +1}{ℑ}_{1}(\frac{\delta_{1}^{1}}{\Psi +1}(\Psi G_{0}\ell_{0}+{ℑ}_{1}G_{0}\ell_{0})+\delta_{2}^{1}G_{0}\ell_{0}+\delta_{3}^{1}\ell_{0})-\ell^{⋆}{\|}^{2}\;\;+(1-\prod_{r=3}^{m}(1-\delta_{3}^{r}))\|\ell_{0}-\ell^{⋆}{\|}^{2}$$

$$=\prod_{r=3}^{m}(1-\delta_{3}^{r})\{\frac{1}{(\Psi +1{)}^{2}}\|\Psi ((\frac{\delta_{1}^{1}}{\Psi +1}(\Psi G_{0}\ell_{0}+{ℑ}_{1}G_{1}\ell_{0}))+\delta_{2}^{1}G_{0}\ell_{0}+\delta_{3}^{1}\ell_{0}\;\;-(\frac{\delta_{1}^{1}}{\Psi +1}(\Psi G_{0}\ell^{⋆}+{ℑ}_{1}G_{0}\ell^{⋆})+\delta_{2}^{1}G_{0}\ell^{⋆}+\delta_{3}^{1}\ell^{⋆}))\;\;+{ℑ}_{1}(\frac{\delta_{1}^{1}}{\Psi +1}(\Psi G_{0}\ell_{0}+{ℑ}_{1}G_{0}\ell_{0})+\delta_{2}^{1}G_{0}\ell_{0}+\delta_{3}^{1}\ell_{0})\;\;-{ℑ}_{1}(\frac{\delta_{1}^{1}}{\Psi +1}(\Psi G_{0}\ell^{⋆}+{ℑ}_{1}G_{0}\ell^{⋆})+\delta_{2}^{1}G_{0}\ell^{⋆}+\delta_{3}^{1}\ell^{⋆}){\|}^{2}\}+(1-\prod_{r=3}^{m}(1-\delta_{3}^{r}))\|\ell_{0}-\ell^{⋆}{\|}^{2}$$

$$\leq \prod_{r=3}^{m}(1-\delta_{3}^{r})\{\frac{1}{(\Psi +1{)}^{2}}[(\Psi +1{)}^{2}\|\frac{\delta_{1}^{1}}{\Psi +1}(\Psi G_{0}\ell_{0}+{ℑ}_{1}G_{0}\ell_{0})+\delta_{2}^{1}G_{0}\ell_{0}+\delta_{3}^{1}\ell_{0}-\ell^{⋆}{\|}^{2}]\}\;\;+(1-\prod_{r=3}^{m}(1-\delta_{3}^{r}))\|\ell_{0}-\ell^{⋆}{\|}^{2}$$

$$=\prod_{r=3}^{m}(1-\delta_{3}^{r})\{\|(1-\delta_{3}^{1})[\frac{\delta_{1}^{1}}{\left(\Psi +1)(1-\delta_{3}^{1}\right)}(\Psi G_{0}\ell_{0}+{ℑ}_{1}G_{0}\ell_{0})+\frac{\delta_{2}^{1}}{\left(1-\delta_{3}^{1}\right)}G_{0}\ell_{0}]+\delta_{3}^{1}\ell_{0}-\ell^{⋆}{\|}^{2}\}\;\;+(1-\prod_{r=3}^{m}(1-\delta_{3}^{r}))\|\ell_{0}-\ell^{⋆}{\|}^{2}$$

$$=\prod_{r=3}^{m}(1-\delta_{3}^{r})\|(1-\delta_{3}^{1})[\frac{\delta_{1}^{1}}{\left(\Psi +1)(1-\delta_{3}^{1}\right)}(\Psi (G_{0}\ell_{0}-\ell^{⋆})+{ℑ}_{1}G_{0}\ell_{0}-\ell^{⋆})\;\;+\frac{\delta_{2}^{1}}{\left(1-\delta_{3}^{1}\right)}(G_{0}\ell_{0}-\ell^{⋆})]+\delta_{3}^{1}(\ell_{0}-\ell^{⋆}){\|}^{2}+(1-\prod_{r=3}^{m}(1-\delta_{3}^{r}))\|\ell_{0}-\ell^{⋆}{\|}^{2}$$

$$\leq \prod_{r=3}^{m}(1-\delta_{3}^{r})\{\frac{\left(1-\delta_{3}^{1}\right)}{(\Psi +1{)}^{2}}\|\frac{\delta_{1}^{1}}{\left(1-\delta_{3}^{1}\right)}(\Psi (G_{0}\ell_{0}-\ell^{⋆})+{ℑ}_{1}G_{0}\ell_{0}-\ell^{⋆})\;\;+\frac{\delta_{2}^{1}}{\left(1-\delta_{3}^{1}\right)}((\Psi +1)(G_{0}\ell_{0}-\ell^{⋆})){\|}^{2}+\delta_{3}^{1}\|\ell_{0}-\ell^{⋆}{\|}^{2}\}+(1-\prod_{r=3}^{m}(1-\delta_{3}^{r}))\|\ell_{0}-\ell^{⋆}{\|}^{2}$$

$$=\prod_{r=3}^{m}(1-\delta_{3}^{r})\{\frac{\left(1-\delta_{3}^{1}\right)}{(\Psi +1{)}^{2}}\|\frac{\delta_{1}^{1}}{\left(1-\delta_{3}^{1}\right)}(\Psi (G_{0}\ell_{0}-\ell^{⋆})+{ℑ}_{1}G_{0}\ell_{0}-\ell^{⋆})\;\;+(1-\frac{\delta_{1}^{1}}{1-\delta_{3}^{1}})((\Psi +1)(G_{0}\ell_{0}-\ell^{⋆})){\|}^{2}+\delta_{3}^{1}\|\ell_{0}-\ell^{⋆}{\|}^{2}\}+(1-\prod_{r=3}^{m}(1-\delta_{3}^{r}))\|\ell_{0}-\ell^{⋆}{\|}^{2}$$

$$=\prod_{r=3}^{m}(1-\delta_{3}^{r})\{\frac{\left(1-\delta_{3}^{1}\right)}{(\Psi +1{)}^{2}}\|\frac{\delta_{1}^{1}}{\left(1-\delta_{3}^{1}\right)}[(\Psi (G_{0}\ell_{0}-\ell^{⋆})+{ℑ}_{1}G_{0}\ell_{0}-\ell^{⋆})-(\Psi +1)(G_{0}\ell_{0}-\ell^{⋆})]\;\;+(\Psi +1)(G_{0}\ell_{0}-\ell^{⋆}){\|}^{2}+\delta_{3}^{1}\|\ell_{0}-\ell^{⋆}{\|}^{2}\}+(1-\prod_{r=3}^{m}(1-\delta_{3}^{r}))\|\ell_{0}-\ell^{⋆}{\|}^{2}$$

$$\leq \prod_{r=3}^{m}(1-\delta_{3}^{r})\{\frac{\left(1-\delta_{3}^{1}\right)}{(\Psi +1{)}^{2}}\{(\Psi +1{)}^{2}\|G_{0}\ell_{0}-\ell^{⋆}{\|}^{2}+2\frac{\delta_{1}^{1}}{1-\delta_{3}^{1}}\langle (\Psi (G_{0}\ell_{0}-\ell^{⋆})+{ℑ}_{1}G_{0}\ell_{0}-\ell^{⋆})\;\;-(\Psi +1)(G_{0}\ell_{0}-\ell^{⋆}),j((\Psi +1)(G_{0}\ell_{0}-\ell^{⋆}))\rangle \;\;+2B^{2}(\frac{\delta_{1}^{1}}{1-\delta_{3}^{1}}{)}^{2}\|(\Psi (G_{0}\ell_{0}-\ell^{⋆})+{ℑ}_{1}G_{0}\ell_{0}-\ell^{⋆})-(\Psi +1)(G_{0}\ell_{0}-\ell^{⋆}){\|}^{2}\}\;\;+\delta_{3}^{1}\|\ell_{0}-\ell^{⋆}{\|}^{2}\}+(1-\prod_{r=3}^{m}(1-\delta_{3}^{r}))\|\ell_{0}-\ell^{⋆}{\|}^{2}$$

$$=\prod_{r=3}^{m}(1-\delta_{3}^{r})\{\frac{\left(1-\delta_{3}^{1}\right)}{(\Psi +1{)}^{2}}\{(\Psi +1{)}^{2}\|G_{0}\ell_{0}-\ell^{⋆}{\|}^{2}+2\frac{\delta_{1}^{1}}{1-\delta_{3}^{1}}\langle \Psi (G_{0}\ell_{0}-\ell^{⋆})\;\;+{ℑ}_{1}G_{0}\ell_{0}-\ell^{⋆},j((\Psi +1)(G_{0}\ell_{0}-\ell^{⋆}))\rangle \;\;+2\frac{\delta_{1}^{1}(\Psi +1{)}^{2}}{1-\delta_{3}^{1}}\langle \ell^{⋆}-G_{0}\ell_{0},j(G_{0}\ell_{0}-\ell^{⋆})\rangle \;\;+2B^{2}(\frac{\delta_{1}^{1}}{1-\delta_{3}^{1}}{)}^{2}\|{ℑ}_{1}G_{0}\ell_{0}-G_{0}\ell_{0}{\|}^{2}\}+\delta_{3}^{1}\|\ell_{0}-\ell^{⋆}{\|}^{2}\}+(1-\prod_{r=3}^{m}(1-\delta_{3}^{r}))\|\ell_{0}-\ell^{⋆}{\|}^{2}$$

$$\leq \prod_{r=3}^{m}(1-\delta_{3}^{r})\{\frac{\left(1-\delta_{3}^{1}\right)}{(\Psi +1{)}^{2}}\{(\Psi +1{)}^{2}\|G_{0}\ell_{0}-\ell^{⋆}{\|}^{2}+2\frac{\delta_{1}^{1}}{1-\delta_{3}^{1}}[(\Psi +1{)}^{2}\|G_{0}\ell_{0}-\ell^{⋆}{\|}^{2}\;\;-\lambda \|(I-{ℑ}_{1})G_{0}\ell_{0}{\|}^{2}]-2\frac{\delta_{1}^{1}(\Psi +1{)}^{2}}{1-\delta_{3}^{1}}\|\ell^{⋆}-G_{0}\ell_{0}{\|}^{2}\;\;+2B^{2}(\frac{\delta_{1}^{1}}{1-\delta_{3}^{1}}{)}^{2}\|{ℑ}_{1}G_{0}\ell_{0}-G_{0}\ell_{0}{\|}^{2}+\delta_{3}^{1}\|\ell_{0}-\ell^{⋆}{\|}^{2}\}\}+(1-\prod_{r=3}^{m}(1-\delta_{3}^{r}))\|\ell_{0}-\ell^{⋆}{\|}^{2}$$

$$=\prod_{r=3}^{m}(1-\delta_{3}^{r})\{\frac{\left(1-\delta_{3}^{1}\right)}{(\Psi +1{)}^{2}}\{(\Psi +1{)}^{2}\|G_{0}\ell_{0}-\ell^{⋆}{\|}^{2}\;\;-2\frac{\delta_{1}^{1}}{1-\delta_{3}^{1}}(\lambda -B^{2}(\frac{\delta_{1}^{1}}{1-\delta_{3}^{1}}))\|(I-{ℑ}_{1})G_{0}\ell_{0}{\|}^{2}\}\;\;+\delta_{3}^{1}\|\ell_{0}-\ell^{⋆}{\|}^{2}\}+(1-\prod_{r=3}^{m}(1-\delta_{3}^{r}))\|\ell_{0}-\ell^{⋆}{\|}^{2}$$

$$\leq \prod_{r=3}^{m}(1-\delta_{3}^{r})[(1-\delta_{3}^{1})\|G_{0}\ell_{0}-\ell^{⋆}{\|}^{2}+\delta_{3}^{1}\|\ell_{0}-\ell^{⋆}{\|}^{2}]+(1-\prod_{r=3}^{m}(1-\delta_{3}^{r}))\|\ell_{0}-\ell^{⋆}{\|}^{2}$$

$$=\prod_{r=2}^{m}(1-\delta_{3}^{r})\|\ell_{0}-\ell^{⋆}{\|}^{2}+(1-\prod_{r=2}^{m}(1-\delta_{3}^{r}))\|\ell_{0}-\ell^{⋆}{\|}^{2}$$

$$=\|\ell_{0}-\ell^{⋆}{\|}^{2}.$$ (2.5)

It therefore follows from (2.5) that

$$\|G_{r}\ell_{0}-\ell^{⋆}{\|}^{2}\leq \|\ell_{0}-\ell^{⋆}{\|}^{2},$$ (2.6)

for every r=1,2,⋯,m. Also, since for t=1,2,⋯,m−1, we obtain, using (2.5), that


$$\|\ell_{0}-\ell^{⋆}{\|}^{2}\leq \prod_{r=t+1}^{m}(1-\delta_{3}^{r})\|G_{t}\ell_{0}-\ell^{⋆}{\|}^{2}+(1-\prod_{r=t+1}^{m}(1-\delta_{3}^{r}))\|\ell_{0}-\ell^{⋆}{\|}^{2}$$

$$=\prod_{r=t+1}^{m}(1-\delta_{3}^{r})\|{ℑ}_{\omega ,t}(\delta_{1}^{t}{ℑ}_{\omega ,t}G_{t-1}+\delta_{2}^{t}G_{t-1}+\delta_{3}^{t}I)\ell_{0}-\ell^{⋆}{\|}^{2}\;\;+(1-\prod_{r=t+1}^{m}(1-\delta_{3}^{r}))\|\ell_{0}-\ell^{⋆}{\|}^{2}$$

$$=\prod_{r=t+1}^{m}(1-\delta_{3}^{r})\|\frac{\Psi }{\Psi +1}(\frac{\delta_{1}^{t}}{\Psi +1}(\Psi G_{t-1}\ell_{0}+{ℑ}_{t}G_{t-1}\ell_{0})+\delta_{2}^{t}G_{t-1}\ell_{0}+\delta_{3}^{t}\ell_{0})\;\;+\frac{1}{\Psi +1}{ℑ}_{t}(\frac{\delta_{1}^{t}}{\Psi +1}(\Psi G_{t-1}\ell_{0}+{ℑ}_{t}G_{t-1}\ell_{0})+\delta_{2}^{t}G_{t-1}\ell_{0}+\delta_{3}^{t}\ell_{0})-\ell^{⋆}{\|}^{2}\;\;+(1-\prod_{r=t+1}^{m}(1-\delta_{3}^{r}))\|\ell_{0}-\ell^{⋆}{\|}^{2}$$

$$=\prod_{r=t+1}^{m}(1-\delta_{3}^{r})\{\frac{1}{(\Psi +1{)}^{2}}\|\Psi ((\frac{\delta_{1}^{t}}{\Psi +1}(\Psi G_{t-1}\ell_{0}+{ℑ}_{t}G_{t-1}\ell_{0}))+\delta_{2}^{t}G_{t-1}\ell_{0}+\delta_{3}^{t}\ell_{0}\;\;-(\frac{\delta_{1}^{t}}{\Psi +1}(\Psi G_{t-1}\ell^{⋆}+{ℑ}_{t}G_{t-1}\ell^{⋆})+\delta_{2}^{t}G_{t-1}\ell^{⋆}+\delta_{3}^{t}\ell^{⋆}))\;\;+{ℑ}_{t}(\frac{\delta_{1}^{t}}{\Psi +1}(\Psi G_{t-1}\ell_{0}+{ℑ}_{t}G_{t-1}\ell_{0})+\delta_{2}^{t}G_{t-1}\ell_{0}+\delta_{3}^{t}\ell_{0})\;\;-{ℑ}_{t}(\frac{\delta_{1}^{t}}{\Psi +1}(\Psi G_{t-1}\ell^{⋆}+{ℑ}_{t}G_{t-1}\ell^{⋆})+\delta_{2}^{t}G_{t-1}\ell^{⋆}+\delta_{3}^{t}\ell^{⋆}){\|}^{2}\}\;\;+(1-\prod_{r=t+1}^{m}(1-\delta_{3}^{r}))\|\ell_{0}-\ell^{⋆}{\|}^{2}$$

$$\leq \prod_{r=t+1}^{m}(1-\delta_{3}^{r})\{\frac{1}{(\Psi +1{)}^{2}}[(\Psi +1{)}^{2}\|\frac{\delta_{1}^{t}}{\Psi +1}(\Psi G_{t-1}\ell_{0}+{ℑ}_{t}G_{t-1}\ell_{0})\;\;+\delta_{2}^{t}G_{t-1}\ell_{0}+\delta_{3}^{t}\ell_{0}-\ell^{⋆}{\|}^{2}]\}+(1-\prod_{i=t+1}^{N}(1-\delta_{3}^{i}))\|\ell_{0}-\ell^{⋆}{\|}^{2}$$

$$=\prod_{r=t+1}^{m}(1-\delta_{3}^{r})\{\|(1-\delta_{3}^{t})[\frac{\delta_{1}^{t}}{\left(\Psi +1)(1-\delta_{3}^{t}\right)}(\Psi G_{t-1}\ell_{0}+{ℑ}_{t}G_{t-1}\ell_{0})+\frac{\delta_{2}^{t}}{\left(1-\delta_{3}^{t}\right)}G_{t-1}\ell_{0}]\;\;+\delta_{3}^{t}\ell_{0}-\ell^{⋆}{\|}^{2}\}+(1-\prod_{r=t+1}^{m}(1-\delta_{3}^{r}))\|\ell_{0}-\ell^{⋆}{\|}^{2}$$

$$=\prod_{r=t+1}^{m}(1-\delta_{3}^{r})\|(1-\delta_{3}^{t})[\frac{\delta_{1}^{t}}{\left(\Psi +1)(1-\delta_{3}^{t}\right)}(\Psi (G_{t-1}\ell_{0}-\ell^{⋆})+{ℑ}_{t}G_{t-1}\ell_{0}-\ell^{⋆})\;\;+\frac{\delta_{2}^{t}}{\left(1-\delta_{3}^{t}\right)}(G_{t-1}\ell_{0}-\ell^{⋆})]+\delta_{3}^{t}(\ell_{0}-\ell^{⋆}){\|}^{2}+(1-\prod_{r=t+1}^{m}(1-\delta_{3}^{r}))\|\ell_{0}-\ell^{⋆}{\|}^{2}$$

$$\leq \prod_{r=t+1}^{m}(1-\delta_{3}^{r})\{\frac{\left(1-\delta_{3}^{t}\right)}{(\Psi +1{)}^{2}}\|\frac{\delta_{1}^{t}}{\left(1-\delta_{3}^{t}\right)}(\Psi (G_{t-1}\ell_{0}-\ell^{⋆})+{ℑ}_{t}G_{t-1}\ell_{0}-\ell^{⋆})\;\;+\frac{\delta_{2}^{t}}{\left(1-\delta_{3}^{t}\right)}((\Psi +1)(G_{t-1}\ell_{0}-\ell^{⋆})){\|}^{2}+\delta_{3}^{t}\|\ell_{0}-\ell^{⋆}{\|}^{2}\}+(1-\prod_{r=t+1}^{m}(1-\delta_{3}^{r}))\|\ell_{0}-\ell^{⋆}{\|}^{2}$$

$$=\prod_{r=t+1}^{m}(1-\delta_{3}^{r})\{\frac{\left(1-\delta_{3}^{t}\right)}{(\Psi +1{)}^{2}}\|\frac{\delta_{1}^{t}}{\left(1-\delta_{3}^{t}\right)}(\Psi (G_{t-1}\ell_{0}-\ell^{⋆})+{ℑ}_{t}G_{t-1}\ell_{0}-\ell^{⋆})\;\;+(1-\frac{\delta_{1}^{t}}{1-\delta_{3}^{t}})((\Psi +1)(G_{t-1}\ell_{0}-\ell^{⋆})){\|}^{2}+\delta_{3}^{t}\|\ell_{0}-\ell^{⋆}{\|}^{2}\}+(1-\prod_{r=t+1}^{m}(1-\delta_{3}^{r}))\|\ell_{0}-\ell^{⋆}{\|}^{2}$$

$$=\prod_{r=t+1}^{m}(1-\delta_{3}^{r})\{\frac{\left(1-\delta_{3}^{t}\right)}{(\Psi +1{)}^{2}}\|\frac{\delta_{1}^{t}}{\left(1-\delta_{3}^{t}\right)}[(\Psi (G_{t-1}\ell_{0}-\ell^{⋆})+{ℑ}_{t}G_{t-1}\ell_{0}-\ell^{⋆})\;\;-(\Psi +1)(G_{t-1}\ell_{0}-\ell^{⋆})]+(\Psi +1)(G_{t-1}\ell_{0}-\ell^{⋆}){\|}^{2}+\delta_{3}^{t}\|\ell_{0}-\ell^{⋆}{\|}^{2}\}\;\;+(1-\prod_{r=t+1}^{m}(1-\delta_{3}^{r}))\|\ell_{0}-\ell^{⋆}{\|}^{2}$$

$$\leq \prod_{r=t+1}^{m}(1-\delta_{3}^{r})\{\frac{\left(1-\delta_{3}^{t}\right)}{(\Psi +1{)}^{2}}\{(\Psi +1{)}^{2}\|G_{t-1}\ell_{0}-\ell^{⋆}{\|}^{2}+2\frac{\delta_{1}^{t}}{1-\delta_{3}^{t}}\langle (\Psi (G_{t-1}\ell_{0}-\ell^{⋆})\;\;+{ℑ}_{t}G_{t-1}\ell_{0}-\ell^{⋆})-(\Psi +1)(G_{t-1}\ell_{0}-\ell^{⋆}),j((\Psi +1)(G_{t-1}\ell_{0}-\ell^{⋆}))\rangle \;\;+2B^{2}(\frac{\delta_{1}^{t}}{1-\delta_{3}^{t}}{)}^{2}\|(\Psi (G_{t-1}\ell_{0}-\ell^{⋆})+{ℑ}_{t}G_{t-1}\ell_{0}-\ell^{⋆})-(\Psi +1)(G_{t-1}\ell_{0}-\ell^{⋆}){\|}^{2}\}\;\;+\delta_{3}^{t}\|\ell_{0}-\ell^{⋆}{\|}^{2}\}+(1-\prod_{r=t+1}^{m}(1-\delta_{3}^{r}))\|\ell_{0}-\ell^{⋆}{\|}^{2}$$

$$=\prod_{r=t+1}^{m}(1-\delta_{3}^{r})\{\frac{\left(1-\delta_{3}^{t}\right)}{(\Psi +1{)}^{2}}\{(\Psi +1{)}^{2}\|G_{t-1}\ell_{0}-\ell^{⋆}{\|}^{2}+2\frac{\delta_{1}^{t}}{1-\delta_{3}^{t}}\langle \Psi (G_{t-1}\ell_{0}-\ell^{⋆})\;\;+{ℑ}_{t}G_{t-1}\ell_{0}-\ell^{⋆},j((\Psi +1)(G_{t-1}\ell_{0}-\ell^{⋆}))\rangle \;\;+2\frac{\delta_{1}^{t}(\Psi +1{)}^{2}}{1-\delta_{3}^{t}}\langle \ell^{⋆}-G_{t-1}\ell_{0},j(G_{0}\ell_{t-1}-\ell^{⋆})\rangle \;\;+2B^{2}(\frac{\delta_{1}^{t}}{1-\delta_{3}^{t}}{)}^{2}\|{ℑ}_{t}G_{t-1}\ell_{0}-G_{t-1}\ell_{0}{\|}^{2}\}+\delta_{3}^{t}\|\ell_{0}-\ell^{⋆}{\|}^{2}\}+(1-\prod_{r=t+1}^{m}(1-\delta_{3}^{r}))\|\ell_{0}-\ell^{⋆}{\|}^{2}$$

$$\leq \prod_{r=t+1}^{m}(1-\delta_{3}^{r})\{\frac{\left(1-\delta_{3}^{t}\right)}{(\Psi +1{)}^{2}}\{(\Psi +1{)}^{2}\|G_{t-1}\ell_{0}-\ell^{⋆}{\|}^{2}+2\frac{\delta_{1}^{t}}{1-\delta_{3}^{t}}[(\Psi +1{)}^{2}\|G_{t-1}\ell_{0}-\ell^{⋆}{\|}^{2}\;\;-\lambda \|(I-{ℑ}_{t})G_{t-1}\ell_{0}{\|}^{2}]-2\frac{\delta_{1}^{t}(\Psi +1{)}^{2}}{1-\delta_{3}^{t}}\|\ell^{⋆}-G_{t-1}\ell_{0}{\|}^{2}\;\;+2B^{2}(\frac{\delta_{1}^{t}}{1-\delta_{3}^{t}}{)}^{2}\|{ℑ}_{t}G_{t-1}\ell_{0}-G_{t-1}\ell_{0}{\|}^{2}+\delta_{3}^{t}\|\ell_{0}-\ell^{⋆}{\|}^{2}\}\}+(1-\prod_{r=t+1}^{m}(1-\delta_{3}^{r}))\|\ell_{0}-\ell^{⋆}{\|}^{2}$$

$$=\prod_{r=t+1}^{m}(1-\delta_{3}^{r})\{\frac{\left(1-\delta_{3}^{t}\right)}{(\Psi +1{)}^{2}}\{(\Psi +1{)}^{2}\|G_{t-1}\ell_{0}-\ell^{⋆}{\|}^{2}\;\;-2\frac{\delta_{1}^{t}}{1-\delta_{3}^{t}}(\lambda -B^{2}(\frac{\delta_{1}^{t}}{1-\delta_{3}^{t}}))\|(I-{ℑ}_{t})G_{t-1}\ell_{0}{\|}^{2}\}\;\;+\delta_{3}^{t}\|\ell_{0}-\ell^{⋆}{\|}^{2}\}+(1-\prod_{r=t+1}^{m}(1-\delta_{3}^{r}))\|\ell_{0}-\ell^{⋆}{\|}^{2},$$

it follows that

$$G_{t-1}\ell_{0}={ℑ}_{t}G_{t-1}\ell_{0}.$$ (2.7)

Using (2.7), we obtain that $\ell_{0}={ℑ}_{1}\ell_{0}$; that is, $\ell_{0}\in F({ℑ}_{1})$. Again, from the definition of ${\text{D}}^{\text{A}}$, we obtain that


$$G_{1}\ell_{0}={ℑ}_{\omega ,1}(\alpha_{1}^{1}{ℑ}_{\omega ,1}G_{0}\ell_{0}+\alpha_{2}^{1}G_{0}\ell_{0}+\alpha_{3}^{1}\ell_{0})$$

$$=((1-\omega )I+\omega {ℑ}_{1})[\alpha_{1}^{1}((1-\omega )\ell_{0}+\omega {ℑ}_{1}G_{0}\ell_{0})+\alpha_{2}^{1}G_{0}\ell_{0}+\alpha_{3}^{1}\ell_{0}]$$

$$=((1-\omega )I+\omega {ℑ}_{1})\ell_{0}=\ell_{0}.$$ (2.8)

(2.5) and (2.8) imply that


$$\|\ell_{0}-\ell^{⋆}{\|}^{2}\leq \prod_{r=3}^{m}(1-\delta_{3}^{r})\{\frac{\left(1-\delta_{3}^{2}\right)}{(\Psi +1{)}^{2}}\{(\Psi +1{)}^{2}\|G_{1}\ell_{0}-\ell^{⋆}{\|}^{2}+2\frac{\delta_{1}^{2}}{1-\delta_{3}^{2}}[(\Psi +1{)}^{2}\|G_{1}\ell_{0}-\ell^{⋆}{\|}^{2}\;\;-\lambda \|(I-{ℑ}_{2})G_{1}\ell_{0}{\|}^{2}]-2\frac{\delta_{1}^{2}(\Psi +1{)}^{2}}{1-\delta_{3}^{2}}\|\ell^{⋆}-G_{1}\ell_{0}{\|}^{2}\;\;+2B^{2}(\frac{\delta_{1}^{2}}{1-\delta_{3}^{2}}{)}^{2}\|{ℑ}_{2}G_{1}\ell_{0}-G_{1}\ell_{0}{\|}^{2}+\delta_{3}^{2}\|\ell_{0}-\ell^{⋆}{\|}^{2}\}\}\;\;+(1-\prod_{r=3}^{m}(1-\delta_{3}^{r}))\|\ell_{0}-\ell^{⋆}{\|}^{2}$$

$$=\prod_{r=3}^{m}(1-\delta_{3}^{r})\{\frac{\left(1-\delta_{3}^{2}\right)}{(\Psi +1{)}^{2}}\{(\Psi +1{)}^{2}\|\ell_{0}-\ell^{⋆}{\|}^{2}\;\;-2\frac{\delta_{1}^{2}}{1-\delta_{3}^{2}}(\lambda -B^{2}(\frac{\delta_{1}^{2}}{1-\delta_{3}^{2}}))\|(I-{ℑ}_{2})\ell_{0}{\|}^{2}\}\;\;+\delta_{3}^{2}\|\ell_{0}-\ell^{⋆}{\|}^{2}\}+(1-\prod_{r=3}^{m}(1-\delta_{3}^{r}))\|\ell_{0}-\ell^{⋆}{\|}^{2}$$

$$=\prod_{r=3}^{m}(1-\delta_{3}^{r})\{\frac{\left(1-\delta_{3}^{2}\right)}{(\Psi +1{)}^{2}}\{(\Psi +1{)}^{2}\|\ell_{0}-\ell^{⋆}{\|}^{2}\;\;-2\frac{\delta_{1}^{2}}{1-\delta_{3}^{2}}(\lambda -B^{2}(\frac{\delta_{1}^{2}}{1-\delta_{3}^{2}}))\|(I-{ℑ}_{2})\ell_{0}{\|}^{2}\}\}\;\;+\prod_{r=3}^{m}(1-\delta_{3}^{r})\delta_{3}^{2}\|\ell_{0}-\ell^{⋆}{\|}^{2}+(1-\prod_{i=3}^{N}(1-\delta_{3}^{i}))\|\ell_{0}-\ell^{⋆}{\|}^{2}$$

$$=\prod_{r=2}^{m}(1-\delta_{3}^{r})\{\|\ell_{0}-\ell^{⋆}{\|}^{2}-2\frac{\delta_{1}^{2}}{1-\delta_{3}^{2}}(\lambda -B^{2}(\frac{\delta_{1}^{2}}{1-\delta_{3}^{2}}))\|(I-{ℑ}_{2})\ell_{0}{\|}^{2}\}\;\;+(1-\prod_{r=2}^{m}(1-\delta_{3}^{r}))\|\ell_{0}-\ell^{⋆}{\|}^{2}$$

$$=\|\ell_{0}-\ell^{⋆}{\|}^{2}-2\prod_{r=2}^{m}(1-\delta_{3}^{r})\frac{\delta_{1}^{2}}{1-\delta_{3}^{2}}(\lambda -B^{2}(\frac{\delta_{1}^{2}}{1-\delta_{3}^{2}}))\|(I-{ℑ}_{2})\ell_{0}{\|}^{2}.$$

The last inequality implies that

$$2\prod_{r=2}^{m}(1-\delta_{3}^{r})\frac{\delta_{1}^{2}}{1-\delta_{3}^{2}}(\lambda -B^{2}(\frac{\delta_{1}^{2}}{1-\delta_{3}^{2}}))\|(I-{ℑ}_{2})\ell_{0}{\|}^{2}\leq 0.$$ (2.9)

Now, since $B^{2}\leq \lambda$, it follows from the properties of $\delta_{1}$ and $\delta_{3}$ that


$$\|(I-{ℑ}_{2})\ell_{0}\|=0,$$

hence ${ℑ}_{2}\ell_{0}=\ell_{0}$.

Using Definition 2.2 and the fact that


$$G_{t-1}\ell_{0}={ℑ}_{t}G_{t-1}\ell_{0},\;for\;t=1,2,\cdots ,m-1,$$

we obtain


$$G_{2}\ell_{0}={ℑ}_{\omega ,2}(\alpha_{1}^{2}{ℑ}_{\omega ,2}G_{1}\ell_{0}+\alpha_{2}^{2}G_{1}\ell_{0}+\alpha_{3}^{2}\ell_{0})$$

$$={ℑ}_{\omega ,2}[\alpha_{1}^{2}((1-\omega )G_{1}\ell_{0}+\omega {ℑ}_{2}G_{1}\ell_{0})+\alpha_{2}^{2}G_{1}\ell_{0}+\alpha_{3}^{2}\ell_{0}]$$

$$={ℑ}_{\omega ,2}[\alpha_{1}^{2}((1-\omega )G_{1}\ell_{0}+\omega G_{1}\ell_{0})+\alpha_{2}^{2}G_{1}\ell_{0}+\alpha_{3}^{2}\ell_{0}]$$

$$={ℑ}_{\omega ,2}[\alpha_{1}^{2}G_{1}\ell_{0}+\alpha_{2}^{2}G_{1}\ell_{0}+\alpha_{3}^{2}\ell_{0}]$$

$$={ℑ}_{\omega ,2}[(1-\alpha_{3}^{2})G_{1}\ell_{0}+\alpha_{3}^{2}\ell_{0}]$$

$$={ℑ}_{\omega ,2}\ell_{0}$$

$$=((1-\omega )I+\omega {ℑ}_{2})\ell_{0}$$

$$=\frac{1}{\Psi +1}(\Psi \ell_{0}+{ℑ}_{2}\ell_{0})$$ (2.10)

and


$$G_{3}\ell_{0}={ℑ}_{\omega ,3}(\alpha_{1}^{3}{ℑ}_{\omega ,3}G_{2}\ell_{0}+\alpha_{2}^{3}G_{2}\ell_{0}+\alpha_{3}^{3}\ell_{0})$$

$$={ℑ}_{\omega ,3}[\alpha_{1}^{3}((1-\omega )G_{2}\ell_{0}+\omega {ℑ}_{3}G_{2}\ell_{0})+\alpha_{2}^{3}G_{2}\ell_{0}+\alpha_{3}^{3}\ell_{0}]$$

$$={ℑ}_{\omega ,3}[\alpha_{1}^{3}((1-\omega )G_{2}\ell_{0}+\omega G_{2}\ell_{0})+\alpha_{2}^{3}G_{2}\ell_{0}+\alpha_{3}^{3}\ell_{0}]$$

$$={ℑ}_{\omega ,3}[\alpha_{1}^{3}G_{2}\ell_{0}+\alpha_{2}^{3}G_{2}\ell_{0}+\alpha_{3}^{3}\ell_{0}]$$

$$={ℑ}_{\omega ,3}[(1-\alpha_{3}^{3})G_{2}\ell_{0}+\alpha_{3}^{3}\ell_{0}]$$

$$=\frac{1}{\Psi +1}(\Psi I+{ℑ}_{3})[(1-\alpha_{3}^{3})G_{2}\ell_{0}+\alpha_{3}^{3}\ell_{0}].$$ (2.11)

From (2.5), (2.10), (2.11) and the uniform convexity of E, we obtain the following:


$$\|\ell_{0}-\ell^{⋆}{\|}^{2}\leq \prod_{r=4}^{m}(1-\delta_{3}^{r})\|G_{3}\ell_{0}-\ell^{⋆}{\|}^{2}+(1-\prod_{r=4}^{m}(1-\delta_{3}^{r}))\|\ell_{0}-\ell^{⋆}{\|}^{2}$$

$$=\prod_{r=4}^{m}(1-\delta_{3}^{r})\|\frac{1}{\Psi +1}(\Psi I+{ℑ}_{3})[(1-\alpha_{3}^{3})G_{2}\ell_{0}+\alpha_{3}^{3}\ell_{0}]-\ell^{⋆}{\|}^{2}\;\;+(1-\prod_{r=4}^{m}(1-\delta_{3}^{r}))\|\ell_{0}-\ell^{⋆}{\|}^{2}$$

$$=\prod_{r=4}^{m}(1-\delta_{3}^{r})\{\frac{1}{(\Psi +1{)}^{2}}\|(\Psi I+{ℑ}_{3})[(1-\alpha_{3}^{3})G_{2}\ell_{0}+\alpha_{3}^{3}\ell_{0}]-\ell^{⋆}{\|}^{2}\}\;\;+(1-\prod_{r=4}^{m}(1-\delta_{3}^{r}))\|\ell_{0}-\ell^{⋆}{\|}^{2}$$

$$\leq \prod_{r=4}^{m}(1-\delta_{3}^{r})\|(1-\alpha_{3}^{3})(G_{2}\ell_{0}-\ell^{⋆})+\alpha_{3}^{3}(\ell_{0}-\ell^{⋆}){\|}^{2}\;\;+(1-\prod_{r=4}^{m}(1-\delta_{3}^{r}))\|\ell_{0}-\ell^{⋆}{\|}^{2}$$

$$=\prod_{r=4}^{m}(1-\delta_{3}^{r})\|(1-\alpha_{3}^{3})(\frac{1}{\Psi +1}(\Psi (\ell_{0}-\ell^{⋆})+{ℑ}_{2}\ell_{0}-\ell^{⋆}))+\alpha_{3}^{3}(\ell_{0}-\ell^{⋆}){\|}^{2}\;\;+(1-\prod_{r=4}^{m}(1-\delta_{3}^{r}))\|\ell_{0}-\ell^{⋆}{\|}^{2}$$

$$\leq \prod_{r=4}^{m}(1-\delta_{3}^{r})\{\frac{\left(1-\alpha_{3}^{3}\right)}{(\Psi +1{)}^{2}}\|\Psi (\ell_{0}-\ell^{⋆})+{ℑ}_{2}\ell_{0}-\ell^{⋆}{\|}^{2}+\alpha_{3}^{3}\|\ell_{0}-\ell^{⋆}{\|}^{2}\;\;-\delta_{1}^{3}(1-\delta_{1}^{3})g(\|{ℑ}_{2}\ell_{0}-\ell_{0}\|)\}+(1-\prod_{r=4}^{m}(1-\delta_{3}^{r}))\|\ell_{0}-\ell^{⋆}{\|}^{2}$$

$$\leq \prod_{r=4}^{m}(1-\delta_{3}^{r})\{(1-\alpha_{3}^{3})\|\ell_{0}-\ell^{⋆}{\|}^{2}+\alpha_{3}^{3}\|\ell_{0}-\ell^{⋆}{\|}^{2}\;\;-\delta_{1}^{3}(1-\delta_{1}^{3})g(\|{ℑ}_{2}\ell_{0}-\ell_{0}\|)\}+(1-\prod_{r=4}^{m}(1-\delta_{3}^{r}))\|\ell_{0}-\ell^{⋆}{\|}^{2}.$$ (2.12)

(2.12) implies that

$$g(\|{ℑ}_{2}\ell_{0}-\ell_{0}\|)=0.$$ (2.13)

Assume that ${ℑ}_{2}\ell_{0}\neq \ell_{0}$, then we have $\|{ℑ}_{2}\ell_{0}-\ell_{0}\|>0$. By the properties of *g*, we have

$$0=g(0)<g(\|{ℑ}_{2}\ell_{0}-\ell_{0}\|)=0.$$ (2.14)

This is a contradiction. Thus, ${ℑ}_{2}\ell_{0}=\ell_{0}$. Using (2.10), we get ${ℑ}_{2}\ell_{0}=\ell_{0}=G_{2}\ell_{0}$.

From Definition 2.2, we obtain $G_{3}\ell_{0}$ as follows:


$$G_{3}\ell_{0}={ℑ}_{\omega ,3}(\alpha_{1}^{3}{ℑ}_{\omega ,3}G_{2}\ell_{0}+\alpha_{2}^{3}G_{2}\ell_{0}+\alpha_{3}^{3}\ell_{0})$$

$$={ℑ}_{\omega ,3}[\alpha_{1}^{3}((1-\omega )G_{2}\ell_{0}+\omega {ℑ}_{3}G_{2}\ell_{0})+\alpha_{2}^{3}G_{2}\ell_{0}+\alpha_{3}^{3}\ell_{0}]$$

$$={ℑ}_{\omega ,3}[\alpha_{1}^{3}((1-\omega )G_{2}\ell_{0}+\omega G_{2}\ell_{0})+\alpha_{2}^{3}G_{2}\ell_{0}+\alpha_{3}^{3}\ell_{0}]$$

$$={ℑ}_{\omega ,3}[\alpha_{1}^{3}G_{2}\ell_{0}+\alpha_{2}^{3}G_{2}\ell_{0}+\alpha_{3}^{3}\ell_{0}]$$

$$={ℑ}_{\omega ,3}[(1-\alpha_{2}^{3})G_{2}\ell_{0}+\alpha_{3}^{3}\ell_{0}]$$

$$={ℑ}_{\omega ,3}\ell_{0}$$

$$=((1-\omega )I+\omega {ℑ}_{3})\ell_{0}$$

$$=\frac{1}{\Psi +1}(\Psi \ell_{0}+{ℑ}_{2}\ell_{0})$$

Using the same approach as in above, we obtain


$${ℑ}_{3}\ell_{0}=\ell_{0}=G_{3}\ell_{0}.$$

By continuing in this manner, we can conclude that

$${ℑ}_{r}\ell_{0}=\ell_{0}=G_{r}\ell_{0},$$ (2.15)

for r=1,2,⋯,m−1. Now, since using (2.5) and (2.15), we obtain


$$\|\ell_{0}-\ell^{⋆}{\|}^{2}\leq \prod_{r=3}^{m}(1-\delta_{3}^{r})\{\frac{\left(1-\delta_{3}^{m}\right)}{(\Psi +1{)}^{2}}\{(\Psi +1{)}^{2}\|G_{N-1}\ell_{0}-\ell^{⋆}{\|}^{2}\;\;+2\frac{\delta_{1}^{m}}{1-\delta_{3}^{m}}[(\Psi +1{)}^{2}\|G_{m-1}\ell_{0}-\ell^{⋆}{\|}^{2}\;\;-\lambda \|(I-{ℑ}_{m})G_{m-1}\ell_{0}{\|}^{2}]-2\frac{\delta_{1}^{m}(\Psi +1{)}^{2}}{1-\delta_{3}^{m}}\|\ell^{⋆}-G_{m-1}\ell_{0}{\|}^{2}\;\;+2B^{2}(\frac{\delta_{1}^{m}}{1-\delta_{3}^{m}}{)}^{2}\|{ℑ}_{2}G_{m-1}\ell_{0}-G_{m-1}\ell_{0}{\|}^{2}+\delta_{3}^{m}\|\ell_{0}-\ell^{⋆}{\|}^{2}\}\}\;\;+(1-\prod_{r=3}^{m}(1-\delta_{3}^{r}))\|\ell_{0}-\ell^{⋆}{\|}^{2}$$

$$=\prod_{r=3}^{m}(1-\delta_{3}^{r})\{\frac{\left(1-\delta_{3}^{m}\right)}{(\Psi +1{)}^{2}}\{(\Psi +1{)}^{2}\|\ell_{0}-\ell^{⋆}{\|}^{2}\;\;-2\frac{\delta_{1}^{m}}{1-\delta_{3}^{m}}(\lambda -B^{2}(\frac{\delta_{1}^{m}}{1-\delta_{3}^{m}}))\|(I-{ℑ}_{m})\ell_{0}{\|}^{2}\}\;\;+\delta_{3}^{m}\|\ell_{0}-\ell^{⋆}{\|}^{2}\}+(1-\prod_{r=3}^{m}(1-\delta_{3}^{r}))\|\ell_{0}-\ell^{⋆}{\|}^{2}$$

$$=\prod_{r=3}^{m}(1-\delta_{3}^{r})\{\frac{\left(1-\delta_{3}^{m}\right)}{(\Psi +1{)}^{2}}\{(\Psi +1{)}^{2}\|\ell_{0}-\ell^{⋆}{\|}^{2}\;\;-2\frac{\delta_{1}^{m}}{1-\delta_{3}^{m}}(\lambda -B^{2}(\frac{\delta_{1}^{m}}{1-\delta_{3}^{m}}))\|(I-{ℑ}_{m})\ell_{0}{\|}^{2}\}\}\;\;+\prod_{r=3}^{m}(1-\delta_{3}^{r})\delta_{3}^{m}\|\ell_{0}-\ell^{⋆}{\|}^{2}+(1-\prod_{r=3}^{m}(1-\delta_{3}^{r}))\|\ell_{0}-\ell^{⋆}{\|}^{2}$$

$$=\prod_{r=2}^{m}(1-\delta_{3}^{r})\{\|\ell_{0}-\ell^{⋆}{\|}^{2}-2\frac{\delta_{1}^{2}}{1-\delta_{3}^{2}}(\lambda -B^{2}(\frac{\delta_{1}^{2}}{1-\delta_{3}^{2}}))\|(I-{ℑ}_{m})\ell_{0}{\|}^{2}\}\;\;+(1-\prod_{r=2}^{m}(1-\delta_{3}^{r}))\|\ell_{0}-\ell^{⋆}{\|}^{2},$$

it follows that

$${ℑ}_{m}\ell_{0}=\ell_{0}.$$ (2.16)

From (2.15) and the definition of ${\text{D}}^{\text{A}}$ in Definition 2.2, we get


$$\ell_{0}=D^{A}\ell_{0}=G_{m}\ell_{0}={ℑ}_{\omega ,m}(\alpha_{1}^{m}{ℑ}_{\omega ,m}G_{m-1}\ell_{0}+\alpha_{2}^{m}G_{m-1}\ell_{0}+\alpha_{3}^{m}\ell_{0})$$

$$={ℑ}_{\omega ,m}[\alpha_{1}^{N}((1-\omega )G_{m-1}\ell_{0}+\omega {ℑ}_{m}G_{m-1}\ell_{0})+\alpha_{2}^{m}G_{m-1}\ell_{0}+\alpha_{3}^{m}\ell_{0}]$$

$$={ℑ}_{\omega ,m}[\alpha_{1}^{m}((1-\omega )G_{m-1}\ell_{0}+\omega G_{m-1}\ell_{0})+\alpha_{2}^{m}G_{m-1}\ell_{0}+\alpha_{3}^{m}\ell_{0}]$$

$$={ℑ}_{\omega ,m}[\alpha_{1}^{m}G_{m-1}\ell_{0}+\alpha_{2}^{m}G_{m-1}\ell_{0}+\alpha_{3}^{m}\ell_{0}]$$

$$={ℑ}_{\omega ,m}[(1-\alpha_{2}^{m})G_{m-1}\ell_{0}+\pi_{3}^{m}\ell_{0}]$$

$$={ℑ}_{\omega ,m}\ell_{0}$$

$$=((1-\omega )I+\omega {ℑ}_{m})\ell_{0}$$

$$=\frac{1}{\Psi +1}(\Psi \ell_{0}+{ℑ}_{m}\ell_{0}),$$

which implies that


$${ℑ}_{m}\ell_{0}=\ell_{0}.$$

Considering the above information, we have the following:

$$\ell_{0}\in {⋂}_{r=1}^{m}F({ℑ}_{r})\;and\;\ell_{0}\in {⋂}_{r=1}^{m}F(G_{r})$$ (2.17)

Now, since


$$G_{t-1}\ell_{0}={ℑ}_{t}G_{t-1}\ell_{0},\;for\;t=1,2,\cdots ,m-1,$$

and $\ell_{0}\in {⋂}_{r=1}^{m}F(G_{r})$, it follows that


$${ℑ}_{t}\ell_{0}=\ell_{0},\;for\;t=1,2,\cdots ,m-1$$

Thus, from (2.16), we get

$$\ell_{0}\in {⋂}_{r=1}^{m}F({ℑ}_{r}).$$ (2.18)

Also, from (2.17) and (2.18), we get

$$\ell_{0}\in {⋂}_{r=1}^{m}F({ℑ}_{r})\cap {⋂}_{i=1}^{m}F({ℑ}_{r}).$$ (2.19)

Consequently, $D^{A}\subseteq \underset{r=1}{\overset{m}{⋂}}F({ℑ}_{r})\cap \underset{r=1}{\overset{m}{⋂}}F({ℑ}_{r})$ It is not difficult to see that

$\underset{r=1}{\overset{m}{⋂}}F({ℑ}_{r})\cap \underset{r=1}{\overset{m}{⋂}}F({ℑ}_{r})\subseteq D^{A}$.

By applying (2.5), it is not difficult to see that the mapping ${\text{D}}^{\text{A}}$ is non-expansive. □

We now prove the following important lemmas to obtain the next results.

**Lemma 2.1.** *Let E be a strictly convex Banach space and C⊂E. Let ${ℑ}_{1}\;and\;{ℑ}_{2}$ be two Ψ-enriched non-expansive mapping (ENM) from C into itself with $F({ℑ}_{1})\cap F({ℑ}_{2})\neq 0\;\;\;/$. Define a mapping W:C⟶C by*

$$W\ell =\pi {ℑ}_{1}\ell +(1-\pi ){ℑ}_{2}\ell ,\forall \ell \in C,$$ (2.20)

*where π∈(0,1) is a constant. Then, W is an ENM and $F(W)=F({ℑ}_{1})\cap F({ℑ}_{2})$.*

*Proof:* For every $\ell ,\varpi^{o}\in C$ and (2.20), we obtain


$$\|W\ell -W\varpi^{o}\|=\|\pi {ℑ}_{1}\ell +(1-\pi ){ℑ}_{2}\ell -(\pi {ℑ}_{1}\varpi^{o}+(1-\pi ){ℑ}_{2}\varpi^{o})\|$$

$$=\|\pi [\Psi (\ell -\varpi^{o})+{ℑ}_{1}\ell -{ℑ}_{1}\varpi^{o}]+(1-\pi )[\Psi (\ell -\varpi^{o})+{ℑ}_{2}\ell -{ℑ}_{2}\varpi^{o}]\;\;-\Psi (\ell -\varpi^{o})\|$$

$$\leq \pi \|\Psi (\ell -\varpi^{o})+{ℑ}_{1}\ell -{ℑ}_{1}\varpi^{o}\|+\|(1-\pi )[\Psi (\ell -\varpi^{o})+{ℑ}_{2}\ell -{ℑ}_{2}\varpi^{o}]\;\;-\Psi (\ell -\varpi^{o})\|$$

$$\leq \pi \|\Psi (\ell -\varpi^{o})+{ℑ}_{1}\ell -{ℑ}_{1}\varpi^{o}\|+(1-\pi )\|\Psi (\ell -\varpi^{o})+{ℑ}_{2}\ell -{ℑ}_{2}\varpi^{o}\|\;\;+\Psi \|\ell -\varpi^{o}\|$$

$$\leq \alpha (\Psi +1)\|\ell -\varpi^{o}\|+(1-\pi )(\Psi +1)\|\ell -\varpi^{o}\|+\Psi \|\ell -\varpi^{o}\|$$

$$=(2\Psi +1)\|\ell -\varpi^{o}\|.$$

Observe that 2Ψ∈[0,∞) whenever Ψ∈[0,∞). Hence, *W* is an *ENM* and $F(W)=F({ℑ}_{1})\cap F({ℑ}_{2})$. □

**Lemma 2.2.** *Let E be a strictly convex Banach space and C⊂E. Let ${ℑ}_{1},{ℑ}_{2}\;and\;{ℑ}_{3}$ be three Ψ-enriched non-expansive mappings from C into itself with $F({ℑ}_{1})\cap F({ℑ}_{2})\cap F({ℑ}_{3})\neq 0\;\;\;/$. Define a mapping W by*


$$W\ell =\pi {ℑ}_{1}\ell +\omega {ℑ}_{2}\ell +\varrho {ℑ}_{3}\ell ,\forall \ell \in C,$$

*where π,ω,ϱ∈(0,1) are constants with π+ω+ϱ=1. Then, W is an ENM and $F(W)=F({ℑ}_{1})\cap F({ℑ}_{2})\cap F({ℑ}_{3})$.*

*Proof:* Using the definition of the mapping *W*, we get


$$W\ell =\pi {ℑ}_{1}\ell +\omega {ℑ}_{2}\ell +\varrho {ℑ}_{3}\ell$$

$$=\pi {ℑ}_{1}\ell +(1-\pi )(\frac{\omega }{1-\pi }{ℑ}_{2}\ell +\frac{\gamma }{1-\pi }{ℑ}_{3}\ell )$$

$$=\pi {ℑ}_{1}\ell +(1-\pi )(\frac{\omega }{1-\pi }{ℑ}_{2}\ell +(1-\frac{\omega }{1-\pi }){ℑ}_{3}\ell )$$

$$=\pi {ℑ}_{1}\ell +(1-\pi )U_{1}\ell ,$$ (2.21)

where $U_{1}=\frac{\omega }{1-\pi }{ℑ}_{2}\;+\;(1\;-\;\frac{\omega }{1-\pi }){ℑ}_{3}$. It therefore follows from Lemma 2.1 that $F(U_{1})=F({ℑ}_{2})\cap F({ℑ}_{3})$ and *U*_1_ is *ENM*. Also, (2.21) and Lemma 2.1 give the conclusion that $F(W)=F({ℑ}_{1})\cap F(U_{1})$ and *W* is *ENM*. Thus, $F(W)=F({ℑ}_{1})\cap F({ℑ}_{2})\cap F({ℑ}_{3})$. □

**Theorem 2.2.** *Let $E,C,Q_{C},A,B,\{{ℑ}_{i}{\}}_{i=1}^{m},(\Psi ,\beta_{i}),\{{ℑ}_{i}{\}}_{i=1}^{m},\delta_{i}$ and ${\text{D}}^{\text{A}}$ be as described in Assumption $\Delta_{1}$. Let $\{\ell_{n}{\}}_{n=1}^{\infty }$ be a sequence developed by $\ell_{1},u\in C$ as*

$$\ell_{n+1}=\pi_{n}u+\omega_{n}\ell_{n}+\varrho_{n}Q_{C}(I-aA)\ell_{n}+\varphi_{n}Q_{C}(I-bB)\ell_{n}+\xi_{n}D^{A}\ell_{n},\forall n\geq 1,$$ (2.22)

*where $\left\{\pi_{n}{\}}_{n=1}^{\infty },\{\omega_{n}{\}}_{n=1}^{\infty },\{\varrho_{n}{\}}_{n=1}^{\infty },\{\varphi_{n}{\}}_{n=1}^{\infty },\{\xi_{n}{\}}_{n=1}^{\infty }\subset [0,1\right]$ with $\pi_{n}+\omega_{n}+\varrho_{n}+\varphi_{n}+\xi_{n}=1$ and satisfying the requirements of Assumption $\Delta_{2}$ Then, $\{\ell_{n}{\}}_{n=1}^{\infty }$ converges strongly to $\nu_{0}=Q_{F}u$, where $Q_{F}$ is the sunny non-expansive retraction of C onto F.*

*Proof:* Since $Q_{C}(I-aA)$ and $Q_{C}(I-bB)$ belong to the class of nonexpansive mappings (see [10] for details), we move on to show that the sequences $\{\ell_{n}{\}}_{n=1}^{\infty },\;\{Q_{C}(I\;-\;aA)\ell_{n}{\}}_{n=1}^{\infty },\;\{Q_{C}(I\;-\;bB)\ell_{n}{\}}_{n=1}^{\infty }\;and\;\{D^{A}\ell_{n}{\}}_{n=1}^{\infty }$ are bounded. To achieve this, let $\ell^{⋆}\in F$. Then, using Lemma 1.3, we obtain that $\ell^{⋆}\in F(Q_{C}(I\;-\;aA)),F(Q_{C}(I\;-\;bB))$. Using (3.1), we get


$$\|\ell_{n+1}-\ell^{⋆}\|=\|\pi_{n}u+\omega_{n}\ell_{n}+\rho_{n}Q_{C}(I-aA)\ell_{n}+\varphi_{n}Q_{C}(I-bB)\ell_{n}+\xi_{n}D^{A}\ell_{n}-\ell^{⋆}\|$$

$$\leq \pi_{n}\|u-\ell^{⋆}\|+\omega_{n}\|\ell_{n}-\ell^{⋆}\|+\rho_{n}\|Q_{C}(I-aA)\ell_{n}-\ell^{⋆}\|\;\;+\varphi_{n}\|Q_{C}(I-bB)\ell_{n}-\ell^{⋆}\|+\xi_{n}\|D^{A}\ell_{n}-\ell^{⋆}\|$$

$$\leq \pi_{n}\|u-\ell^{⋆}\|+\omega_{n}\|\ell_{n}-\ell^{⋆}\|+\rho_{n}\|\ell_{n}-\ell^{⋆}\|+\varphi_{n}\|\ell_{n}-\ell^{⋆}\|\;\;+\xi_{n}\|\ell_{n}-\ell^{⋆}\|$$

$$=\pi_{n}\|u-\ell^{⋆}\|+(1-\pi_{n})\|\ell_{n}-\ell^{⋆}\|$$

$$\leq \max\{\|u-\ell^{⋆}\|,\|\ell_{n}-\ell^{⋆}\|\}.$$

Using inductional hypothesis, we get


$$\|\ell_{n}-\ell^{⋆}\|\leq \max\{\|u-\ell^{⋆}\|,\|\ell_{1}-\ell^{⋆}\|\}.$$

Hence, the sequence $\{\ell_{n}{\}}_{n=1}^{\infty }$ is bounded. As a consequence, the sequences

$\{Q_{C}(I-aA)\ell_{n}{\}}_{n=1}^{\infty },\{Q_{C}(I-bB)\ell_{n}{\}}_{n=1}^{\infty }\;and\;\{D^{A}\ell_{n}{\}}_{n=1}^{\infty }$ are also bounded.

Next, we prove that $\lim_{n\to \infty }\|\ell_{n+1}-\ell_{n}\|=0$. Using (3.1), we obtain the following estimates;


$$\|\ell_{n+1}-\ell_{n}\|=\|\pi_{n}u+\omega_{n}\ell_{n}+\rho_{n}Q_{C}(I-aA)\ell_{n}+\varphi_{n}Q_{C}(I-bB)\ell_{n}+\xi_{n}D^{A}\ell_{n}\;\;-[\pi_{n-1}u+\omega_{n-1}\ell_{n-1}+\rho_{n-1}Q_{C}(I-aA)\ell_{n-1}+\varphi_{n-1}Q_{C}(I-bB)\ell_{n-1}\;\;+\xi_{n-1}D^{A}\ell_{n-1}]\|$$

$$\leq |\pi_{n}-\pi_{n-1}|\|u\|+\omega_{n}\|\ell_{n}-\ell_{n-1}\|+|\omega_{n}-\omega_{n-1}|\|\ell_{n-1}\|\;\;+\rho_{n}\|Q_{C}(I-aA)\ell_{n}-Q_{C}(I-aA)\ell_{n-1}\|+|\rho_{n}-\rho_{n-1}|\|Q_{C}(I-aA)\ell_{n-1}\|\;\;+\varphi_{n}\|Q_{C}(I-bB)\ell_{n}-Q_{C}(I-bB)\ell_{n-1}\|+|\varphi_{n}-\varphi_{n-1}|\|Q_{C}(I-bB)\ell_{n-1}\|\;\;+\xi_{n}\|D^{A}wp_{n}-D^{A}\ell_{n-1}\|+|\xi_{n-1}-\xi_{n}|\|D^{A}\ell_{n}\|$$

$$\leq (1-\pi_{n})\|\ell_{n}-\ell_{n-1}\|+|\pi_{n}-\pi_{n-1}|\|u\|+|\omega_{n}-\omega_{n-1}|\|\ell_{n-1}\|\;\;+|\rho_{n}-\rho_{n-1}|\|Q_{C}(I-aA)\ell_{n-1}\|+|\varphi_{n}-\varphi_{n-1}|\|Q_{C}(I-bB)\ell_{n}\|\;\;+|\xi_{n-1}-\xi_{n}|\|D^{A}\ell_{n}\|.$$ (2.23)

(2.23) and Lemma 1.6 imply that

$$\lim_{n\to \infty }\|\ell_{n+1}-\ell_{n}\|=0.$$ (2.24)

Next, we prove that

$$\lim_{n\to \infty }\|Q_{C}(I-aA)\ell_{n}-\ell_{n}\|=\lim_{n\to \infty }\|Q_{C}(I-bB)\ell_{n}-\ell_{n}\|=\lim_{n\to \infty }\|D^{A}\ell_{n}-\ell_{n}\|=0$$ (2.25)

Using (3.1), we obtain


$$\|\ell_{n+1}-\ell^{⋆}{\|}^{2}=\|\pi_{n}(u-\ell^{⋆})+\omega_{n}(\ell_{n}-\ell^{⋆})+\rho_{n}(Q_{C}(I-aA)\ell_{n}-\ell^{⋆})\;\;+\varphi_{n}(Q_{C}(I-bB)\ell_{n}-\ell^{⋆})+\xi_{n}(D^{A}\ell_{n}-\ell^{⋆}){\|}^{2}$$

$$=\|\omega_{n}(\ell_{n}-\ell^{⋆})+\rho_{n}(Q_{C}(I-aA)\ell_{n}-\ell^{⋆})+(\pi_{n}+\varphi_{n}+\xi_{n})(\frac{\pi_{n}(u-\ell^{⋆})}{\pi_{n}+\varphi_{n}+\xi_{n}}\;\;+\frac{\varphi_{n}(Q_{C}(I-bB)\ell_{n}-\ell^{⋆})}{\pi_{n}+\varphi_{n}+\xi_{n}}+\frac{\Psi_{n}(D^{A}\ell_{n}-\ell^{⋆})}{\pi_{n}+\varphi_{n}+\xi_{n}}){\|}^{2}$$

$$=\|\omega_{n}(\ell_{n}-\ell^{⋆})+\rho_{n}(Q_{C}(I-aA)\ell_{n}-\ell^{⋆})+\nu_{n}s_{n}{\|}^{2},$$ (2.26)

where $\nu_{n}=\pi_{n}+\varphi_{n}+\xi_{n}$ and $s_{n}=\frac{\pi_{n}(u-\ell^{⋆})}{\pi_{n}+\varphi_{n}+\xi_{n}}+\frac{\varphi_{n}(Q_{C}(I-bB)\ell_{n}-\ell^{⋆})}{\pi_{n}+\varphi_{n}+\xi_{n}}+\frac{\xi_{n}(D^{A}\ell_{n}-\ell^{⋆})}{\pi_{n}+\varphi_{n}+\xi_{n}}$.

Since (2.26) and Lemma 1.2 imply


$$\|\ell_{n+1}-\ell^{⋆}{\|}^{2}\leq \omega_{n}\|\ell_{n}-\ell^{⋆}{\|}^{2}+\rho_{n}\|Q_{C}(I-aA)\ell_{n}-\ell^{⋆}{\|}^{2}+\nu_{n}\|s_{n}{\|}^{2}\;\;-\omega_{n}\rho_{n}g(\|\ell_{n}-Q_{C}(I-aA)\ell_{n}\|)$$

$$\leq (\omega_{n}+\rho_{n})\|\ell_{n}-\ell^{⋆}{\|}^{2}+\nu_{n}(\frac{\pi_{n}\|u-\ell^{⋆}{\|}^{2}}{\pi_{n}+\varphi_{n}+\xi_{n}}+\frac{\varphi_{n}\|Q_{C}(I-bB)\ell_{n}-\ell^{⋆}\|}{\pi_{n}+\varphi_{n}+\xi_{n}}\;\;+\frac{\xi_{n}\|D^{A}\ell_{n}-\ell^{⋆}{\|}^{2}}{\pi_{n}+\varphi_{n}+\xi_{n}})-\omega_{n}\gamma_{n}g(\|\ell_{n}-Q_{C}(I-aA)\ell_{n}\|)$$

$$\leq (\omega_{n}+\rho_{n})\|\ell_{n}-\ell^{⋆}{\|}^{2}+\pi_{n}\|u-\ell^{⋆}{\|}^{2}+\varphi_{n}\|Q_{C}(I-bB)\ell_{n}-\ell^{⋆}\|\;\;+\xi_{n}\|D^{A}\ell_{n}-\ell^{⋆}{\|}^{2}-\omega_{n}\rho_{n}g(\|\ell_{n}-Q_{C}(I-aA)\ell_{n}\|)$$

$$\leq (\omega_{n}+\rho_{n})\|\ell_{n}-\ell^{⋆}{\|}^{2}+\pi_{n}\|u-\ell^{⋆}{\|}^{2}+\varphi_{n}\|\ell_{n}-\ell^{⋆}\|\;\;+\xi_{n}\|\ell_{n}-\ell^{⋆}{\|}^{2}\omega_{n}\rho_{n}-g(\|\ell_{n}-Q_{C}(I-aA)\ell_{n}\|)$$

$$=\|\ell_{n}-\ell^{⋆}{\|}^{2}+\pi_{n}\|u-\ell^{⋆}{\|}^{2}-\omega_{n}\gamma_{n}g(\|\ell_{n}-Q_{C}(I-aA)\ell_{n}\|),$$

it follows that

$$\omega_{n}\rho_{n}g(\|\ell_{n}-Q_{C}(I-aA)\ell_{n}\|)\leq \|\ell_{n}-\ell^{⋆}{\|}^{2}-\|\ell_{n+1}-\ell^{⋆}{\|}^{2}\;\;+\pi_{n}\|u-\ell^{⋆}{\|}^{2}.$$ (2.27)

Observe that


$$\|\ell_{n}-\ell^{⋆}{\|}^{2}=\|\ell_{n}-\ell_{n+1}+\ell_{n+1}-\ell^{⋆}{\|}^{2}$$

$$=\|\ell_{n}-\ell_{n+1}{\|}^{2}+2\|\ell_{n}-\ell_{n+1}\|\|\ell_{n+1}-\ell^{⋆}\|+\|\ell_{n+1}-\ell^{⋆}{\|}^{2}$$

so that

$$\|\ell_{n}-\ell^{⋆}{\|}^{2}-\|\ell_{n+1}-\ell^{⋆}{\|}^{2}=(\|\ell_{n}-\ell_{n+1}\|+2\|\ell_{n+1}-\ell^{⋆}\|)\|\ell_{n}-\ell_{n+1}\|.$$ (2.28)

(2.27) and (2.28) imply that

$$\omega_{n}\rho_{n}g(\|\ell_{n}-Q_{C}(I-aA)\ell_{n}\|)\leq (\|\ell_{n}-\ell_{n+1}\|+2\|\ell_{n+1}-\ell^{⋆}\|)\|\ell_{n}-\ell_{n+1}\|\;\;+\pi_{n}\|u-\ell^{⋆}{\|}^{2}.$$ (2.29)

From (2.24), (2.29) and condition (a), we have


$$\lim_{n\to \infty }g(\|\ell_{n}-Q_{C}(I-aA)\ell_{n}\|)=0,$$

which by employing properties of *g* yields

$$\lim_{n\to \infty }\|\ell_{n}-Q_{C}(I-aA)\ell_{n}\|=0.$$ (2.30)

By using similar argument as in (2.30), we obtain

$$\lim_{n\to \infty }\|\ell_{n}-Q_{C}(I-bB)\ell_{n}\|=\lim_{n\to \infty }\|\ell_{n}-D^{A}\ell_{n}\|=0.$$ (2.31)

Define

$$V\ell =\alpha D^{A}\ell +\beta Q_{C}(I-aA)\ell +\gamma Q_{C}(I-bB)\ell ,$$ (2.32)

for all ℓ∈C with α+β+γ=1. Lemma 2.2 implies that $F(V)=F(Q_{C}(I-aA\cap F(Q_{C}(I-bB))\cap F(D^{A})$ while Lemma 1.3 and Theorem 2.1 imply that $F=V=\underset{r=1}{\overset{m}{⋂}}F({ℑ}_{i})\cap \underset{r=1}{\overset{m}{⋂}}F({ℑ}_{i})\cap S(C,A)\cap S(C,B)$. Using (3.1), we obtain


$$\|V\ell_{n}-\ell_{n}\|\leq \alpha \|D^{A}\ell_{n}-\ell_{n}\|+\beta \|Q_{C}(I-aA)\ell_{n}-\ell_{n}\|+\gamma \|Q_{C}(I-bB)\ell_{n}-\ell_{n}\|,$$

which by (2.31) gives

$$\lim_{n\to \infty }\|V\ell_{n}-\ell_{n}\|=0.$$ (2.33)

(2.33) and Lemma 1.5 imply that

$${lim sup}_{n\to \infty }\langle u-\nu_{0},j(\ell_{n}-\nu_{0})\rangle \leq 0,$$ (2.34)

where $\nu_{0}=Q_{F}u$.

Lastly, we show that the sequence $\{\ell_{n}{\}}_{n=1}^{\infty }$ converges strongly to $\nu_{0}=Q_{F}u$.


$$\|\ell_{n+1}-\nu_{0}{\|}^{2}=\|\pi_{n}(u-\nu_{0})+\omega_{n}(\ell_{n}-\nu_{0})+\rho_{n}(Q_{C}(I-aA)\ell_{n}-\nu_{0})\;\;+\varphi_{n}(Q_{C}(I-bB)\ell_{n}-\ell^{⋆})+\xi_{n}(D^{A}\ell_{n}-\nu_{0}){\|}^{2}$$

$$=\|\pi_{n}(u-\nu_{0})+(1-\pi_{n})(\frac{\omega_{n}(\ell_{n}-\nu_{0})}{1-\pi_{n}}+\frac{\rho_{n}}{\left(1-\pi_{n}\right)}(Q_{C}(I-aA)\ell_{n}-\nu_{0})\;\;+\frac{\varphi_{n}(Q_{C}(I-bB)\ell_{n}-\nu_{0})}{1-\pi_{n}}+\frac{\xi_{n}(D^{A}\ell_{n}-\nu_{0})}{1-\pi_{n}}){\|}^{2}$$

$$\leq \|(1-\pi_{n})(\frac{\omega_{n}(\ell_{n}-\nu_{0})}{1-\pi_{n}}+\frac{\rho_{n}}{\left(1-\pi_{n}\right)}(Q_{C}(I-aA)\ell_{n}-\nu_{0})\;\;+\frac{\varphi_{n}(Q_{C}(I-bB)\ell_{n}-\nu_{0})}{1-\pi_{n}}+\frac{\xi_{n}(D^{A}\ell_{n}-\nu_{0})}{1-\pi_{n}}){\|}^{2}+2\pi_{n}\langle u-\nu_{0},j(\ell_{n+1}-\nu_{0})\rangle$$

$$\leq (1-\pi_{n})\|\ell_{n}-\nu_{0}{\|}^{2}+2\pi_{n}\langle u-\nu_{0},j(\ell_{n+1}-\nu_{0})\rangle .$$ (2.35)

(2.35), condition (a) and Lemma 1.6 imply that $\lim_{n\to \infty }\|\ell_{n}-\nu_{0}\|=0$, and the proof is completed □

## 3 Application

The following strong convergence theorems could be obtained from our main results in the setup of real Banach space. In the sequel, we need the following lemma which is an immediate consequence of Theorem 2.1 and Definition 1.4.

**Lemma 3.1.** *Let $E,C,Q_{C},\{{ℑ}_{i}{\}}_{i=1}^{m},(\Psi ,\beta_{i}),\delta_{i}$ and ${\text{D}}^{\text{A}}$ be as described in Assumption $\Delta_{1}$. Let D be an Ψ- enriched ${\text{S}}^{\text{A}}$-mapping developed from the sequences $\{{ℑ}_{i}{\}}_{i=1}^{m}$ and $\{\delta_{i}{\}}_{i=1}^{m}$. Then, $F(S^{A})={⋂}_{i=1}^{N}F({ℑ}_{i})$ and D is nonexpansive.*

**Theorem 3.1.** *Let $E,C,Q_{C},A,B,\{{ℑ}_{i}{\}}_{i=1}^{m},\delta_{i}$ and ${\text{D}}^{\text{A}}$ be as described in Assumption $\Delta_{1}$. Let $\{\ell_{n}{\}}_{n=1}^{\infty }$ be a sequence developed by $\ell_{1},u\in C$ as*

$$\ell_{n+1}=\pi_{n}u+\omega_{n}\ell_{n}+\rho_{n}Q_{C}(I-aA)\ell_{n}+\varphi_{n}Q_{C}(I-bB)\ell_{n}+\xi_{n}D^{A}\ell_{n},\forall n\geq 1,$$ (3.1)

*where $\left\{\pi_{n}{\}}_{n=1}^{\infty },\{\omega_{n}{\}}_{n=1}^{\infty },\{\rho_{n}{\}}_{n=1}^{\infty },\{\varphi_{n}{\}}_{n=1}^{\infty },\{\xi_{n}{\}}_{n=1}^{\infty }\subset [0,1\right]$ with $\pi_{n}+\omega_{n}+\rho_{n}+\varphi_{n}+\xi_{n}=1$ and satisfying the requirements of Assumption $\Delta_{2}$ Then, $\{\ell_{n}{\}}_{n=1}^{\infty }$ converges strongly to $\nu_{0}=Q_{F}u$, where $Q_{F}$ is the sunny non-expansive retraction of C onto F.*

*Proof:* Set $I={ℑ}_{1}={ℑ}_{2}={ℑ}_{3}=\cdots ={ℑ}_{m}$ in Theorem 2.2. Then, the result follows immediately from Lemma 3.1 and Theorem 2.2. □

**Theorem 3.2.** *Let $E,C,Q_{C},A,B,\{{ℑ}_{i}{\}}_{i=1}^{m}$ and $\delta_{i}$ be as described in Assumption $\Delta_{1}$. Define a mapping $V_{i}:C⟶C$ by $Q_{C}(I-\lambda_{i}A_{i})\ell =V_{i}\ell$, where $\lambda_{i}\in (0,\frac{\alpha_{i}}{B^{2}})$ and B is the 2-uniformly smooth constant of E, for all ℓ∈C and for all i=1,2,⋯,m. Let ${\text{S}}^{\text{A}}$ be an Ψ- enriched ${\text{D}}^{\text{A}}$-mapping developed from the sequences $\{{ℑ}_{i}{\}}_{i=1}^{m}$ and $\{\delta_{i}{\}}_{i=1}^{m}$. Let $\{\ell_{n}{\}}_{n=1}^{\infty }$ be a sequence developed by $\ell_{1},u\in C$ as*

$$\ell_{n+1}=\pi_{n}u+\omega_{n}\ell_{n}+\rho_{n}Q_{C}(I-aA)\ell_{n}+\varphi_{n}Q_{C}(I-bB)\ell_{n}+\xi_{n}D^{A}\ell_{n},\forall n\geq 1,$$ (3.2)

*where $\left\{\pi_{n}{\}}_{n=1}^{\infty },\{\omega_{n}{\}}_{n=1}^{\infty },\{\rho_{n}{\}}_{n=1}^{\infty },\{\varphi_{n}{\}}_{n=1}^{\infty },\{\xi_{n}{\}}_{n=1}^{\infty }\subset [0,1\right]$ with $\pi_{n}+\omega_{n}+\rho_{n}+\varphi_{n}+\xi_{n}=1$ and satisfying the requirements of Assumption $\Delta_{2}$ Then, $\{\ell_{n}{\}}_{n=1}^{\infty }$ converges strongly to $\nu_{0}=Q_{F}u$, where $Q_{F}$ is the sunny non-expansive retraction of C onto F.*

*Proof:* It is easy to see, by using similar argument as in the one used in [10] in an attempt to show that $Q_{C}(I-aA)\ell$ is non-expansive, that $\{V_{i}{\}}_{i=1}^{N}$ is non-expansive mapping. Since from Lemma 1.3 $F(V_{i})=S(C,A_{i})$, for all i=1,2,⋯,m, the conclusion of the proof follows directly from Theorem 2.2. □

## 4 Conclusions

In this study, the idea of Ψ-enriched ${𝐷}^{A}$ mapping is introduced in the setup of a real Banach spaces which generalizes several well-known and vital mappings in the literature. We demonstrated the existence of fixed points of Ψ-enriched ${𝐷}^{A}$ mapping in the setting of 2-uniformly smooth and uniformly convex Banach space. Furthermore, we obtained a common element of finite families of $\beta_{i}$-enriched non-expansive mappings, and $\left(\Psi ,\beta_{i}\right)$-enriched strictly psuedocontractive mappings and variational inequality problems by using newly defined mapping and Mann-Halpern type iterative method. Several previously known results are obtained by our main results. The results of this paper gave an affirmative answer to Question 1.1.
